# Endogenous LXR signaling controls pulmonary surfactant homeostasis and prevents lung inflammation

**DOI:** 10.1007/s00018-024-05310-3

**Published:** 2024-07-06

**Authors:** Irene Hernández-Hernández, Juan V. De La Rosa, Patricia Martín-Rodríguez, Mercedes Díaz-Sarmiento, Carlota Recio, Borja Guerra, Leandro Fernández-Pérez, Theresa E. León, Rosa Torres, Joan Font-Díaz, Angela Roig, Fernando de Mora, Lisardo Boscá, Mario Díaz, Annabel F. Valledor, Antonio Castrillo, Carlos Tabraue

**Affiliations:** 1 Unidad de Biomedicina (Unidad Asociada al CSIC), Instituto Universitario de Investigaciones Biomédicas y Sanitarias (IUIBS), Universidad de Las Palmas de Gran Canaria, Las Palmas de Gran Canaria, Spain; 2 Departamento de Morfología, Universidad de Las Palmas de Gran Canaria, Las Palmas de Gran Canaria, Spain; 3 Departamento de Bioquímica y Biología Molecular, Fisiología, Genética e Inmunología, Universidad de Las Palmas de Gran Canaria, Las Palmas de Gran Canaria, Spain; 4 Instituto de Investigaciones Biomédicas Sols-Morreale (IIBM), CSIC-UAM, Madrid, Spain; 5 Department of Cell Biology, Physiology and Immunology, School of Biology, University of Barcelona, Barcelona, Spain; 6 Institute of Biomedicine of the University of Barcelona (IBUB), Barcelona, Spain; 7 Department of Pharmacology, Therapeutics and Toxicology, Universitat Autònoma de Barcelona, Bellaterra, Spain; 8 Instituto Universitario de Investigaciones Biomédicas y Sanitarias (IUIBS), Farmacología Molecular y Traslacional, Universidad de Las Palmas de Gran Canaria, Las Palmas de Gran Canaria, Spain; 9 Centro de Investigación Biomédica en Red de Enfermedades Cardiovasculares (CIBERCV), Av. Monforte de Lemos 3-5, P-11, Madrid, 28029 Spain; 10 Laboratory of Membrane Physiology and Biophysics, School of Physics, Faculty of Sciences, University of La Laguna, San Cristóbal de La Laguna, Tenerife Spain

**Keywords:** LXR, Alveolar macrophage, Type 2 pneumocyte, Surfactant, Inflammation, Lipidosis

## Abstract

Lung type 2 pneumocytes (T2Ps) and alveolar macrophages (AMs) play crucial roles in the synthesis, recycling and catabolism of surfactant material, a lipid/protein fluid essential for respiratory function. The liver X receptors (LXR), LXRα and LXRβ, are transcription factors important for lipid metabolism and inflammation. While LXR activation exerts anti-inflammatory actions in lung injury caused by lipopolysaccharide (LPS) and other inflammatory stimuli, the full extent of the endogenous LXR transcriptional activity in pulmonary homeostasis is incompletely understood. Here, using mice lacking LXRα and LXRβ as experimental models, we describe how the loss of LXRs causes pulmonary lipidosis, pulmonary congestion, fibrosis and chronic inflammation due to defective *de novo* synthesis and recycling of surfactant material by T2Ps and defective phagocytosis and degradation of excess surfactant by AMs. LXR-deficient T2Ps display aberrant lamellar bodies and decreased expression of genes encoding for surfactant proteins and enzymes involved in cholesterol, fatty acids, and phospholipid metabolism. Moreover, LXR-deficient lungs accumulate foamy AMs with aberrant expression of cholesterol and phospholipid metabolism genes. Using a house dust mite aeroallergen-induced mouse model of asthma, we show that LXR-deficient mice exhibit a more pronounced airway reactivity to a methacholine challenge and greater pulmonary infiltration, indicating an altered physiology of LXR-deficient lungs. Moreover, pretreatment with LXR agonists ameliorated the airway reactivity in WT mice sensitized to house dust mite extracts, confirming that LXR plays an important role in lung physiology and suggesting that agonist pharmacology could be used to treat inflammatory lung diseases.

**Graphical Abstract:**

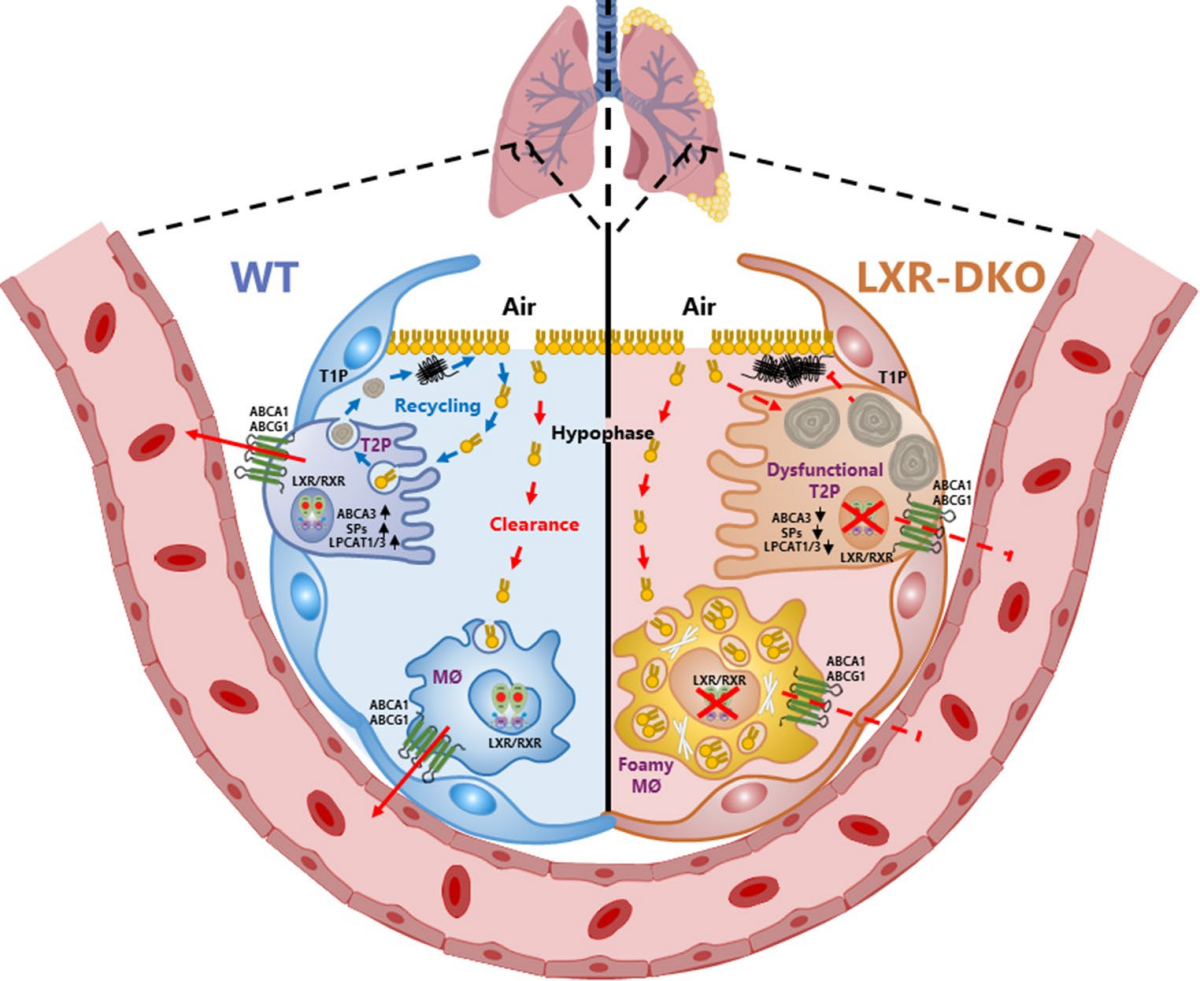

**Supplementary Information:**

The online version contains supplementary material available at 10.1007/s00018-024-05310-3.

## Introduction

The proper functioning of the lung primarily relies on three key cell types: type I (T1P) and type II (T2P) pneumocytes and alveolar macrophages (AM). These cells play crucial roles in facilitating efficient gas exchange and ensuring adequate host defense. While the alveolar epithelium, composed of T1P and T2P, lines the lung’s airspaces and forms the pulmonary parenchyma, AMs contribute to the configuration of the pulmonary stroma, supporting the alveolar epithelium. T1P, which covers approximately 90% of the alveolar surface in adult lungs, is a squamous epithelial cell that closely interacts with the endothelial cells of pulmonary capillaries, facilitating gas exchange. AMs and T2Ps are responsible for synthesizing, recycling, and catabolizing surfactant material, a lipid-rich substance crucial for proper lung function [1, 2].

Pulmonary surfactant serves a dual function by forming the air-liquid interface of the alveolar wall, reducing surface tension to prevent alveolar collapse during the ventilatory cycle, and contributing to pulmonary host defense by opsonizing microbial pathogens. It primarily consists of neutral lipids (mainly cholesterol) and phospholipids, accounting for 90% of the composition, along with four surfactant proteins: surfactant proteins A (SP-A), B (SP-B), C (SP-C), and D (SP-D), comprising the remaining 10% [3]. T2Ps synthesize and secrete surfactant to the alveolar surface, where it is subsequently reabsorbed for recycling and new synthesis. T2Ps are cuboidal epithelial cells characterized by cytoplasmic inclusions known as lamellar bodies, which store protein- and lipid-rich secretory molecules that serve as surfactant material precursors [4]. Moreover, T2Ps also play a significant role in the pulmonary inflammatory response, recruiting monocytes and macrophages through the release of antimicrobial factors, cytokines, and chemokines, thereby influencing the extent and duration of the innate immune response [3, 4].

AMs, as the resident innate immune cells of the respiratory tract, account for 90–95% of the cells collected via bronchoalveolar lavage (BAL). They are well-known for their role in maintaining lung homeostasis by phagocytosing microbes, dead cells, and airborne particles, thereby preventing unnecessary inflammation [5]. Besides modulating immune responses, AMs phagocytose and degrade excess surfactant on the alveolar surface to maintain lung homeostasis. Insufficient secretion of growth factors, such as granulocyte-macrophage colony-stimulating factor (GM-CSF), can impair AM differentiation and lead to defective surfactant clearance, resulting in the accumulation of proteins and phospholipids in the airspaces, known as pulmonary alveolar proteinosis [6]. AMs also contribute to tissue damage control and resolution of inflammation by clearing extracellular debris and apoptotic cells from the airways and producing anti-inflammatory cytokines. Impaired macrophage phagocytic function and apoptosis can contribute to severe pulmonary pathologies associated with chronic inflammatory lung disease [7, 8].

Liver X receptors (LXRs), specifically LXRα and LXRβ, are members of the nuclear receptor superfamily activated by oxysterols and involved in the regulation of lipid metabolism [9]. While LXRβ is ubiquitously expressed in almost all tissues, LXRα is primarily expressed in the liver, spleen, macrophages, intestine, kidney, adipose tissue and lung. LXRs act as intracellular sensors of cholesterol levels and exert important effects on the metabolism of phospholipids, which, along with cholesterol, are major components of cell membranes [10]. Elevation of the cholesterol level in macrophages due to the excessive uptake of lipoproteins or cellular debris, activates LXRs, leading to the induction of a transcriptional program that promotes cholesterol utilization. A representative pathway of this program involves the transcriptional induction of ATP-binding cassette family members, such as ABCA1 and ABCG1, which facilitate the export of cholesterol and phospholipids outside the cell [11]. Additionally, LXRs participate in other transcriptional aspects of macrophage biology, including the inflammatory response, macrophage survival, apoptotic cell clearance, modulation of adaptive immune responses, and the development of a specific macrophage population in the spleen [12].

Previous studies have demonstrated the downregulation of LXR expression during lung inflammation and injury caused by lipopolysaccharides (LPS) and other stimuli [13, 14]. However, the full extent of the LXR signaling pathway’s role in lung histophysiology and its potential alterations in lung pathology are not completely elucidated. In this study, we utilized mice lacking both LXRα and LXRβ as experimental models to uncover an unexpected role of LXRs in lung homeostasis, involving T2Ps and AMs, through the regulation of surfactant synthesis, recycling, and catabolism. Furthermore, our results provide evidence that LXR deficiency promotes chronic pulmonary inflammation, impairing proper lung function.

## Materials and methods

### Experimental animals

Mice on a mixed C57BL6/Sv129 background of different genotypes, WT, Knock-out for *Lxrα* (Nr1h3 -/-), for *Lxrβ* (Nr1h2-/-) or deficient for both genes (*Lxrαβ*-/-, denoted as LXR-DKO) were originally provided by David Mangelsdorf (U. Texas Southwestern, USA) [15]. In some experiments we used LXR-DKO and WT mice backcrossed in C57BL/6 background for more than 10 generations. Csf1r-EGFP (MacGreen) transgenic mice in which the enhanced green fluorescent protein (EGFP) expression sequence has been inserted into the 5’ end of the macrophage colony-stimulating factor receptor (CSF-1 receptor) (Csf1r) gene were originally provided by David Hume [16].

All mice were fed with a standard diet and maintained under pathogen-free conditions, in a temperature-controlled room and a 12-hour light-dark cycle in the animal facilities of Universidad Las Palmas de Gran Canaria (ULPGC) and Parc Científic de Barcelona (PCB). Animals used for experimentation were treated according to ethical standards for animal handling and supervised by the animal ethics research committee of the ULPGC (protocol OEBA-ULPGC 47/2020 R1 and resolution 1237/2021), the Autonomous University of Barcelona (approved by the Generalitat de Catalunya, DAAM9800) and PCB (approved by the Generalitat de Catalunya, DAAM8094).

### MLE-12 cell line

MLE-12 (murine lung epithelial cells, ATTC® CRL-2110™) cell line was cultured in Dulbecco´s Modified Eagle´s medium (DMEM) / F12 medium (Lonza) supplemented with ITS (0.005 mg/ml insulin, 0.01 mg/ml transferrin, 30 nM sodium selenite) (Gibco), 10 nM hydrocortisone (Sigma), 10 nM β-estradiol (Sigma), 10 mM HEPES, 1 mM L-glutamine, (Lonza), penicillin (100U/ml) (Sigma), streptomycin (100 µg/ml) (Sigma) and 2% FBS (Gibco). Before treatment with synthetic ligands, LXR agonist GW3965 (1µM) (Glaxo SmithKline) and LXR antagonist GSK1440233A (denoted here as GW233) (1µM) (Glaxo SmithKline), the cells were washed with PBS and fresh medium containing 0.2% FBS was added.

### Mouse peritoneal macrophages

Thioglycolate-elicited peritoneal macrophages were obtained from mice through intraperitoneal injection of 3 ml of 3% sterile thioglycolate (BD Difco). After 3 days, mice were sacrificed and cells were isolated from the peritoneum by sequential washing with 10 mL of cold phosphate-buffered saline (PBS) and filtered through a 70 μm-pore nylon cell separator (BD FalconTM, BD Bioscience). Cells were cultured in Dulbecco´s Modified Eagle´s medium (DMEM, Lonza) supplemented with 10% fetal bovine serum (Gibco), penicillin (100U/ml) (Sigma) and streptomycin (100 µg/ml) (Sigma).

### Pneumocytes cultures

T2Ps were obtained from mice using an adapted version of the previously established protocols [17, 18]. Mice were anesthetized by intraperitoneal injection with the anesthetic cocktail (pentobarbital 50 mg/kg/ and heparin). First, the pulmonary artery was cannulated and 0.1 mM PBS-EDTA was injected using a heparinized syringe to completely remove blood from the lungs. The trachea of the mice was then cannulated and sutured, and the heart-lung-trachea block was carefully removed from the thorax. Subsequently, the lungs were insufflated with 2.5 ml of enzyme solution, Dispase, 50 U/ml (BD) in Hanks´ Balanced Salt Solution (HBSS, Sigma), and placed in a conical tube containing 1 ml of enzyme solution. After incubation for 6 min at 37 °C, the lungs are cleaned of any remaining non-pulmonary tissue and mechanically homogenized in 3 ml of DNAase solution (50 U/ml in DMEM) using a GentleMACS Dissociator™. The homogenate is then clarified by centrifugation, the pellet is resuspended in DMEM + FBS 10% and filtered by passing through a successive nylon filter (70 and 40 μm pore). The resulting cell suspension was centrifuged at 200*g* for 8 min, the pellet was resuspended in DMEM + FBS 10%, and the cells were seeded onto an IgG-coated surface culture. After incubation for 30 min at 37ºC, the last step is repeated one more time to increase the efficiency of the isolation. Finally, 1 × 10^6^ non-adherent cells were incubated with purified rat anti-mouse CD16/CD32 (Mouse BD Fc Block™, BD) for 5 min at 4ºC before incubating with an anti‐mouse CD326 1:100 (epithelial cell adhesion molecule, EpCAM) Biotin (eBioscience) for 15 min at 4ºC. Subsequently, cells were washed with autoMACS™ running buffer (Miltenyi) and incubated with anti-biotin microbeads 1:10 (Miltenyi) for 15 min. After a new washing, the cell suspension was subjected to positive magnetic separation using a MACS® separation column. Finally, eluted T2Ps were seeded onto Matrigel™-coated surface culture containing DMEM + 10% FBS. T2P cells were identified by assessing the purities of cytocentrifuged cells with a cell-specific antibody for the exclusive marker of T2Ps, anti-pro-SP-C.

### Bronchoalveolar lavage (BAL)

After mice were sacrificed, bronchoalveolar lavage (BAL) was obtained by sequential injection of 5 × 350 µl of sterile PBS-EDTA (0.01 µM) containing 2% foetal bovine serum, through a tracheal catheter and recovering it by gentle aspiration after 30 s. The BAL collected was filtered (70 μm) and centrifuged (500 g, 5 min, 4ºC). The supernatant (BAL fluid, BALF) was used for lipid analysis and lipidosis induction assay. Cell pellet (BAL cells) was resuspended in PBS for trypan blue cell counting in a Neubauer chamber and seed on culture chamber slides precoated with poly-lysine (50 µg/ml) and fibronectin (50 µg/ml), alone or together with LipidTOX™ Red phospholipidosis detection reagent (Invitrogen), 1:2000. After overnight incubation, the cells were washed several times with PBS and fixed with 4% PFA containing Hoechst 33,342 dye (Sigma), 1:5000, for 30 min. Finally, the cells were washed with PBS and incubated with LipidTOX™ Green neutral lipid stain (Invitrogen), 1:1000, for 1 h and mounted with mounting medium (Vectashield Mounting Medium, Vector Laboratories). All slides were analyzed with a Lasser LSM 5 Pa scanning microscope using the LSM 5 Image Examiner program or with an Eclipse Nikon 90i (Nikon). Alternatively, recovered fluids were subjected to cytospin (Shandon CytoSpin III Cytocentrifuge) at 600 rpm for 2 min before May-Grumwald Giemsa or Oil Red-O staining (Sigma) to count at least 2 × 10^3^ cells in randomly selected fields.

### Lipidosis induction assay

Peritoneal macrophages (4 × 10^5^ cells/well) and MLE-12 cells (1.5 × 10^5^ MLE-12 cells/well) were cultured for 24 h on culture chamber slides, precoated with FBS for MLE-12 cells. Subsequently, the cells were incubated with bronchoalveolar lavage fluid (BALF) from WT and LXR-DKO mice, according to an adapted version of the previously established [19]. After overnight incubation with BALF, the cells were washed several times with PBS and fixed with 4% PFA containing a 1:5000 dilution of Hoechst 33,342 dye for 30 min. Finally, the cells were washed with PBS and incubated with LipidTOX™ Green neutral lipid stain 1:1000 in PBS for 1 h and mounted with mounting medium (Vectashield Mounting Medium, Vector Laboratories). All slides were analyzed and counted at least 1 × 10^3^ cells in randomly selected fields with a Lasser LSM 5 PASCAL scanning microscope using the LSM 5 Image Examiner program or with an Eclipse Nikon 90i (Nikon).

### Histology

After perfusion of the lungs with PBS and injection of OCT™ compound medium (Tissue-Tek® Sakura) through a tracheal catheter, lung tissue was collected directly from the animal, embedded in OCT™ compound medium (Tissue-Tek® Sakura), and snap frozen in isopentane at -80ºC. 4 μm frozen sections were air dried, fixed with 4% PFA in PBS for 30 min, (Tissue-Tek® Cryo3®, Sakura) and subjected to histochemistry: H&E, Oil-Red-O, Masson’s trichrome and periodic acid-Schiff (PSA) staining were purchased from Sigma.

### Immunohistochemistry

Frozen sections were air dried, fixed with 4% PFA in PBS for 30 min, permeabilized with 0.1% Triton X-100 (Sigma) in PBS, blocked with 6% bovine serum albumin (BSA) and 2% preimmune serum in PBS, and stained with fluorescence-conjugated antibodies diluted in blocking solution; cell nuclei were stained with DAPI (Vectashield mountain medium fluorescence with DAPI, Vector laboratories). Alternatively, after blocking endogenous tissue peroxidase, non-fluorophore-conjugated antibodies and biotinylated secondary antibodies were visualized by the streptavidin-biotin-peroxidase method (VECTASTAI6®Universal Elite® ABC Kit, Vector Laboratories). Staining was performed with the chromogen 3–3′-diaminobenzidine tetrahydrochloride (DAB) or alkaline phosphatase (DAB-HRP or Vector Blue Substrate Kits, Vector Laboratories). Cell nuclei were counterstained with hematoxilin. All tissue sections were analyzed with a Lasser LSM 5 Pa scanning microscope using the LSM 5 Image Examiner program or with an Eclipse Nikon 90i (Nikon).

### Immunocytochemistry

BAL cells and T2P were cultured for 24 h on culture chamber slides, precoated with poly-lysine (50 µg/ml) and fibronectin (50 µg/ml) for BAL cells and with Corning® Matrigel® Basement Membrane Matrix (Merck) for T2P. After overnight incubation, the cells were washed several times with PBS and fixed with 4% PFA in PBS for 15 min, permeabilized with 0.1% Triton X-100 in PBS, blocked with 6% bovine serum albumin (BSA) and 2% preimmune serum in PBS. Cells were then stained with fluorescence-conjugated antibodies diluted in blocking solution, alone or together with BODIPY® 493/503 fluorescent probe stain (Molecular Probes) to stain neutral lipids green; cell nuclei were stained with DAPI (Vectashield mountain medium fluorescence with DAPI, Vector). Slides were analyzed with a Lasser LSM 5 Pa scanning microscope using the LSM 5 Image Examiner program or with an Eclipse Nikon 90i (Nikon).

### Electron-microscopy procedures

LXR wild-type and DKO mice were sacrificed and perfused with fixation solution (2% glutaraldehyde). Lungs were fixed with 2% OsO4, dehydrated and embedded in epoxy resin. Ultra-thin sections were made with an ultramicrotome (Reichert Ultracut’s Leica) and stained with uranyl acetate and lead citrate and observed with a Zeiss EM 910 transmission electron microscope (Carl ZEISS, Germany) at the ULPGC electron microcopy core facility.

### Bone marrow transplant studies

Bone marrow transplants were performed as detailed before [20]. Briefly, recipient wild-type and LXR-DKO mice (6–8 weeks of age) were sub-lethally irradiated with 900 rads and transplanted with 1.0 × 10^7^ bone marrow cells from 6 to 8 week-old donors (wild-type or LXR-DKO) or Csf1r-eGFP mice by retro-orbital injection. Recipient mice were sacrificed at 16 weeks after transplantation and tissue sections were then analyzed for histology, lipid accumulation and the presence of macrophages by immunofluorescence as detailed above.

### RNA and protein analysis

Total RNA was extracted from tissues or cultured cells using TRIZOL® reagent (Invitrogen) according to the product specifications. The RNA pellet was resuspended with DEPC-treated water, and 1 µg was used for retrotranscription with the iScript cDNA Synthesis kit™ (Bio-Rad) according to the manufacturer’s instructions. For RT-qPCR assay, 5µL of cDNA (2.5 µg) was mixed with 15µL of 2X PCR MasterMix (Diagenode), fluorescein isothiocyanate (FITC) and 0.3 µM qPCR primer mix. Fluorescence emission was detected using a CFX connect™ thermal cycler (Bio-Rad). Relative levels of RNA were calculated using the ΔΔCt method, and individual expression data were normalized to 36B4 gene expression. Primer sequences are displayed in Table S1.

For Western blot analysis, total cell protein extracts from homogenized tissues or cultured cells were resolved by polyacrylamide gel electrophoresis (SDS-PAGE), transferred to PVDF membranes (Bio-Rad), blocked in TBS-T solution containing 5% milk, and incubated with specific primary antibodies recognizing γ-H2AX (Biolegend, 2F3), p53 (Cell Signaling Technology, 7F5), active caspase-1 (Santa Cruz Biotechnology, Sc-514), and GAPDH (Sigma, G9545). After washing and incubation with appropriate horseradish peroxidase-conjugated anti-mouse or anti-rabbit, reactive bands were detected using Clarity Western ECL Substrate® (Bio-Rad) and visualized using the Bio-Rad Chemi-Doc imaging system. Antibodies used for Western Blot analysis are displayed in Table S2.

### Lipid analysis

Pulmonary lipids and BALF surfactant lipids were determined as described [21]. Briefly, total lipids were extracted with chloroform/methanol (2:1, v/v) containing 0.01% butylated hydroxytoluene (BHT) as an antioxidant. The organic solvent was evaporated under a nitrogen stream and the lipid content was determined gravimetrically. Lipid classes were separated by one-dimensional double development high-performance thin-layer chromatography (HPTLC) using methyl acetate/isopropanol/chloroform/methanol/0.25% (w/v) KCl (25:25:25:10:9, v/v) as the developing solvent system for polar lipid classes and hexane/diethyl ether/acetic acid (80:20:2, v/v) as the developing solvent system for neutral lipid classes. Lipid classes were quantified by scanning densitometry using a Shimadzu CS-9001PC dual-wavelength flying spot scanner.

### Sensitization to house dust mite aeroallergens

Mice were exposed to a purified house dust mite (HDM) extract (provided by Alk-Abelló, Spain) with a low lipopolysaccharide content (< 0.5 EU/dose, as determined by the Endosafe LAL Assay, Charles River Laboratories). The HDM extract was administered daily intranasally (i.n.) at a dose of 25 µg/mouse in a volume of 35 µl for 10 consecutive days under isoflurane-induced anaesthesia. Non-sensitized (control) animals were manipulated identically except that they received i.n. saline instead of the HDM extract. In some experiments, LXR activation was induced by i.p. injection of GW3965 (20 mg/kg) 24 h prior to the first HDM administration and throughout the whole course of sensitization. Control mice were injected i.p. with vehicle (DMSO in PBS).

### Assessment of airway reactivity after HDM sensitization

Airway reactivity was analyzed in all animals 24 h after the last exposure to HDM using an invasive technique to measure airway resistance with two Finepointe Series RC sites (Buxco Europe) as described [6, 7]. In brief, mice were anaesthetized with an i.p. injection of ketamine/xylazine. The trachea was exposed and cannulated. The mice were mechanically ventilated at 120 breaths/min and tidal volumes of 12.5 ml/Kg. Baseline readings for resistance were recorded, and increasing doses of aerosolised methacholine were administered. The average of the maximum response for each dose was then calculated.

### Lung histological analysis and determination of inflammation score

Lungs were fixed in formaldehyde at room temperature. After fixation, the lungs were washed in cold PBS. Each lung was cut into three pieces and embedded in paraffin. Sections (10 μm thick) were generated with a microtome and recovered on glass slides treated for increased adhesion (Starfrost, Waldemar Knittel Glassbearbeitungs). The sections were stained with hematoxylin-eosin and mounted with DPX (Sigma). The number and size of inflammatory cell infiltrates was calculated on every slice. For this, photographs were obtained with an Olympus PM10SP Automatic Photomicrography System coupled to a light microscope. Each perivascular and/or peribronchoalveolar inflammatory cell infiltrate was photographed at 10x. The area of each infiltrate was measured (in mm^2^) using the Image J tool “polygonal selection”. For each mouse, the lung inflammation score was calculated by multiplying the average number of infiltrates by the mean infiltrate area.

### Statistical analysis

Data are presented as mean ± standard error (SEM). All determinations were performed in triplicate or quadruplicate and the data shown are representative results from at least three independent experiments or otherwise indicated. Results were analyzed using Prism 8.0 software (GraphPad). Statistical differences between means were tested using Student’s t-test for two groups or analysis of variance (ANOVA) and Kruskal-Wallis for multiple group comparisons. Results with *P* < 0.05 were considered statistically significant. In Fig. 9d, in order to make two independent experiments comparable, the data were normalized using the following procedure. The intensity of each experiment (i.e.) was calculated by determining the mean value of airway resistance between the readings at baseline-PBS and the readings at the two highest doses of methacholine. This mean was calculated using the entire group of animals included in the experiment. The intensities of separate experiments were then normalized by the mean intensity value of all the experiments (im) and, in each experiment, the values of resistance of all mice were multiplied by the normalization factor (im/ie).

## Results

### LXR deficiency alters lung morphology

**Fig. 1:**
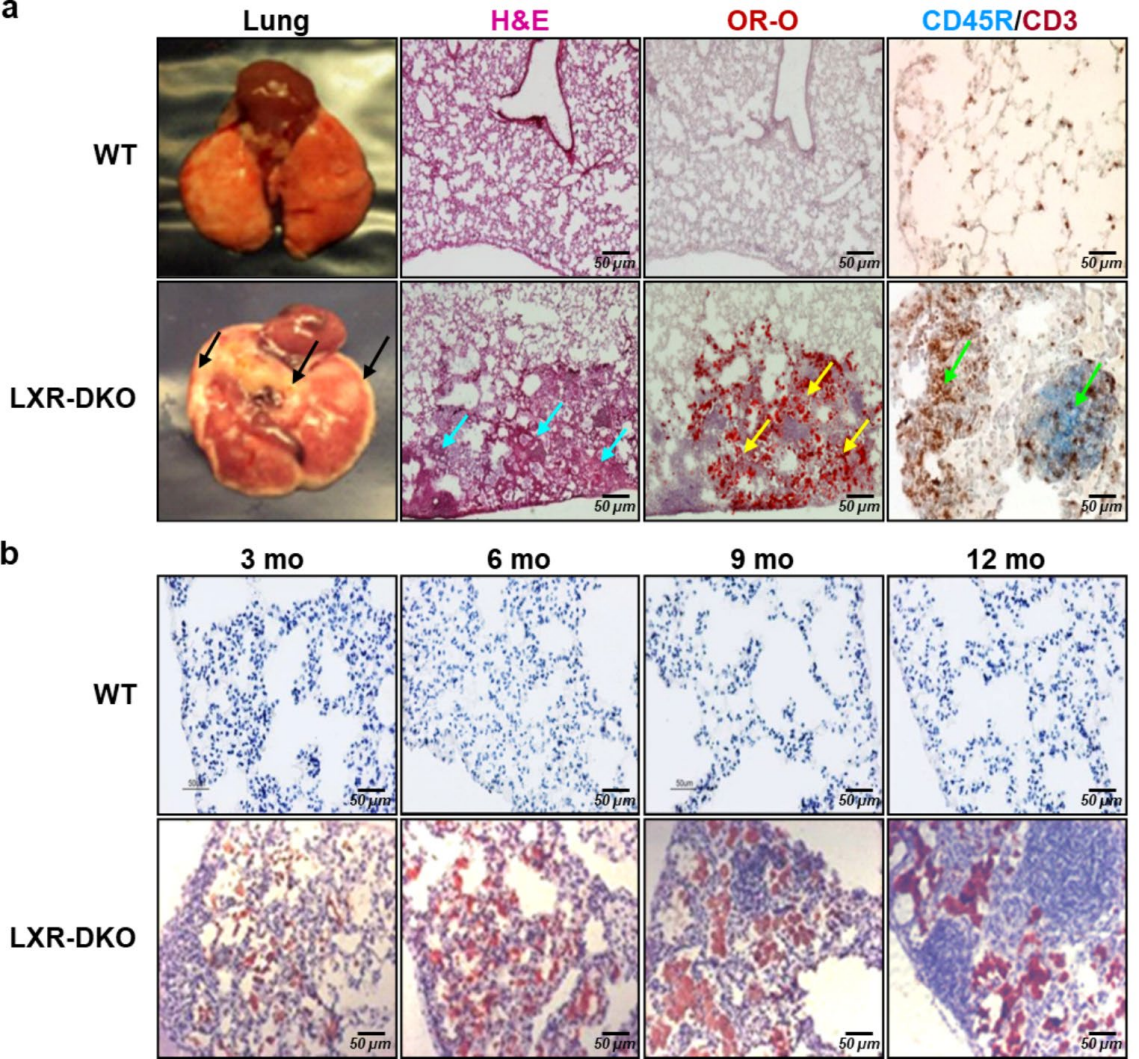
**Lung histopathology in LXR-deficient mice**. **a**: WT or LXR-DKO lungs from 6-month-old mice were subjected to macroscopic analysis by H&E and oil red O (OR-O) staining, and CD45R/CD3 enzymatic immunohistochemistry. Arrows indicate lipid accumulation (black arrows), tissue congestion (blue arrows), neutral lipid accumulation (yellow arrows), and B- and T-lymphocyte infiltration (green arrows). **b**: WT or LXR-DKO lungs at 3, 6, 9 and 12 months of age were subjected to OR-O staining. Scale bars of all images are 50 μm. A representative image of 6 mice per genotype is shown

To determine the importance of LXR signaling in pulmonary histophysiology, we initially carried out a comparative histological analysis of the lung morphology from WT and *Lxr* double knockout (*Nr1h3*^*−/−*^*/Nr1h2*^*−/−*^) mice which will be described here as LXR-DKO mice. Macroscopically, we observed a remarkable pale halo bordering the pulmonary lobes (subpleural zone) in LXR-DKO mice, suggestive of lipid accumulation, which is evident at 6 months of animal life (Fig. 1a, lung; brown arrows). A closer histological analysis of lung areas from LXR-DKO mice by hematoxylin and eosin (H&E) staining revealed abundant cellular infiltrates indicative of tissue congestion (Fig. 1a, H&E; blue arrows). To check for possible lipid accumulation in these areas of the lung, we examined consecutive sections by Oil Red-O staining (OR-O). The appearance of OR-O positive areas (Fig. 1a, OR-O; yellow arrows) revealed an extensive accumulation of neutral lipids between the areas of cellular infiltration, showing that LXR deficiency leads to the development of lipidosis in the lung. Simultaneously, we evaluated the presence of immune cells in the subpleural zone by performing enzymatic immunohistochemistry for lymphocyte markers CD45R and CD3 which revealed areas of cellular congestion in the LXR-DKO lungs with increased B and T lymphocytes compared to LXR-WT lungs (Fig. 1A, CD45R/CD3; green arrows).

Next, we studied the evolution of morphological changes in the lungs of LXR-DKO mice throughout their life, starting at 3 months of age as the first adult age control group. As shown in Fig. 1b, the appearance of OR-O positive regions and accumulation of immune cellular infiltrates is progressive and increases with age in 3- to 12-month-old LXR-DKO mice compared to LXR-WT. Furthermore, deletion of either LXRα or LXRβ had minimal impact on lung histology in 3- and 9-months-old mice, indicating that both LXR isoforms are required to prevent the development of lung lipidosis and damage (Fig.S1). Taken together, these results demonstrate that loss of both LXR isoforms resulted in altered lung morphology characterized by age-dependent development of lipidosis and infiltration of immunocompetent cells, alterations characteristic of an inflammatory phenotype.

### Alveolar macrophage and type II pneumocytes are involved in lung histopathology in LXR DKO mice

**Fig. 2:**
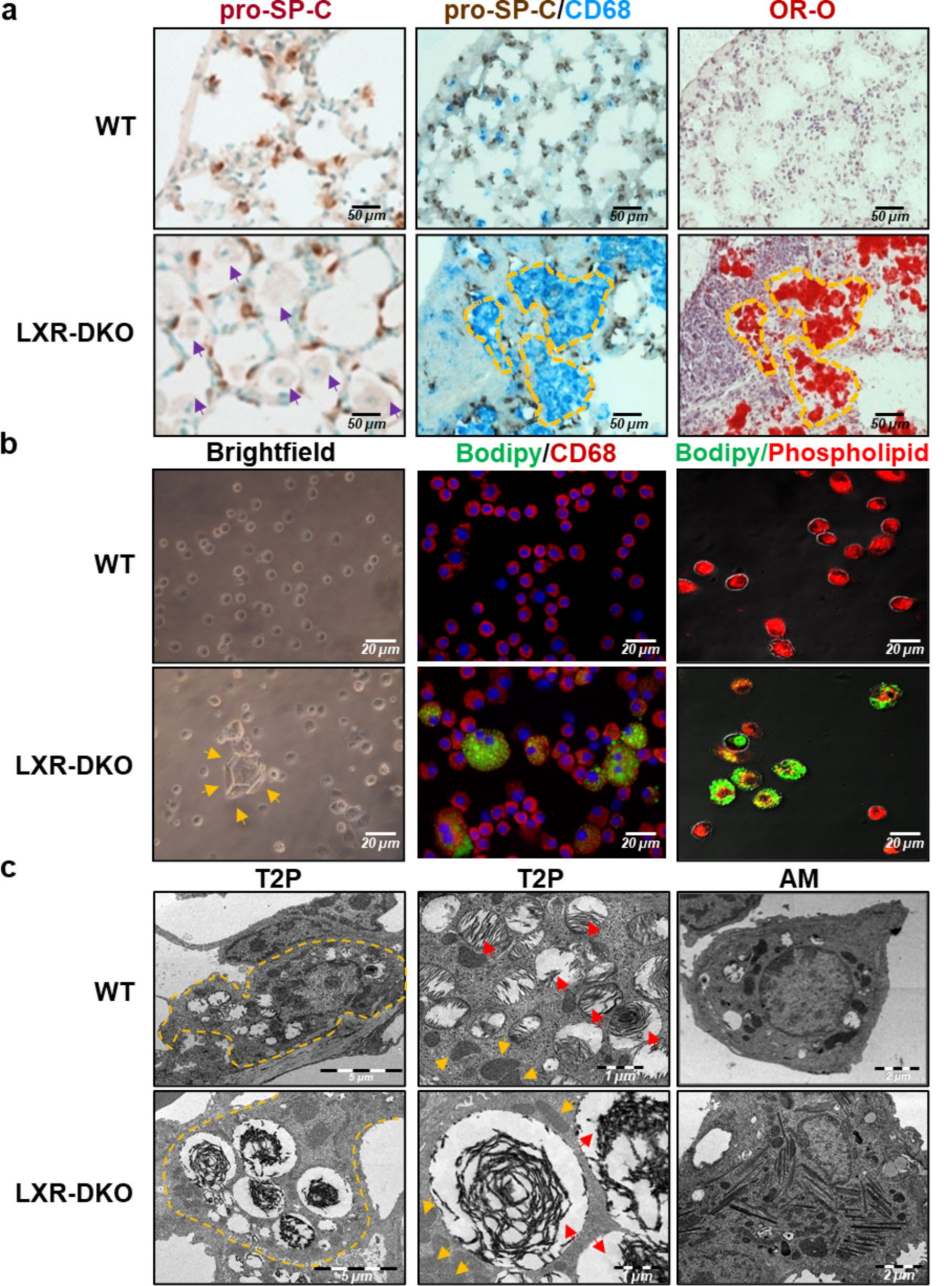
**Disrupted alveolar physiology in LXR-deficient lungs**. **a**: Consecutive sections of lungs from 6-month-old WT or LXR-DKO mice were subjected to enzymatic immunohistochemistry for the detection of pro-SP-C (left panels), double detection of pro-SP-C and CD-68 (middle panels) or Oil Red O (ORO-O) staining (right panels). Scale bars, 50 μm. Purple arrowheads indicate AMs and areas marked with yellow dashed line indicate coinciding areas with regions occupied by AMs in consecutive sections. A representative image of 5 mice per genotype is shown. **b**: BAL cells from WT or LXR-DKO 6-month-old mice was subjected to contrast-phase brightfield microscopy (left panels), Bodipy 493/503 fluorescent probe staining (neutral lipids) together with CD68 immunodetection (middle panels), and Bodipy 493/503 together with phospholipid fluorescent probe staining (right panels). Scale bars, 20 μm. Orange arrowheads indicate large hexagonal extracellular crystals. A representative image of 4 mice per genotype is shown. **c**: Lungs from 6-month-old WT or LXR-DKO mice were subjected to ultrastructural analysis by electron microscopy. Left panels, T2P (8.000 X; Scale bar: 5 μm), areas marked with yellow dashed line indicate cell contours; middle panels, T2P (16.000 X, Scale bar: 1 μm), orange arrowheads indicate mitochondria and red arrowheads indicate lamellar bodies; and right panels, AM (8.000 X, Scale bar: 2 μm). A representative image of 3 mice per genotype is shown

Pneumocytes and AMs are the main cells forming the lung parenchyma architecture. To elucidate whether these cells are involved in the histological disorder of LXR-DKO lungs, T2Ps and AMs were analyzed by pro-SP-C (surfactant protein-C) and CD68 expression, respectively. Pro-SP-C immunodetection (Fig. 2a) revealed a similar number of T2Ps between WT and LXR-DKO mice in contrast to AMs (identified by their characteristic morphology and location, purple arrowheads) which appear to be increased in LXR-DKO lungs. Double immunodetection of pro-SP-C and CD68 (Fig. 2a) confirmed the augmented frequency of the AM population in LXR-DKO lungs. In addition, OR-O positive areas were detected in LXR-DKO lungs, which coincided with regions occupied by AMs (Fig. 2a; yellow dashed line). These results suggest that the morphological impairment and lipidosis developed in LXR-DKO lungs were due to a large increase in the number of foamy macrophages in the alveoli.

We then investigated whether this alteration could be reflected in the phenotypic characteristics of the BAL cell population from LXR-DKO lungs. BAL cells were analyzed by brightfield and confocal fluorescence microscopy using fluorescent probes. Unexpectedly, we observed through brightfield microscopy the presence of large hexagonal extracellular crystals (orange arrowheads) in the BAL (Fig. 2b) from LXR-DKO mice. These crystals resemble those observed in animal models with pulmonary lipidosis and inflammation, such as in Abcg1 -/- mice and whose biochemical nature could respond to the formation of extracellular complex lipoprotein aggregates [22, 23]. Additionally, the identification of cholesterol droplets by using Bodipy 493/503 fluorescent probe together with immunodetection of AM (CD68+) (Fig. 2b) revealed a large increase in the level of cholesterol accumulated by AMs in BAL from LXR-DKO mice. These data suggest that the origin of these foamy cells could be due to the accumulation of cholesterol in the surfactant material and/or the inability of macrophages to efflux intracellular cholesterol in the LXR-DKO lungs.

**Fig. 3:**
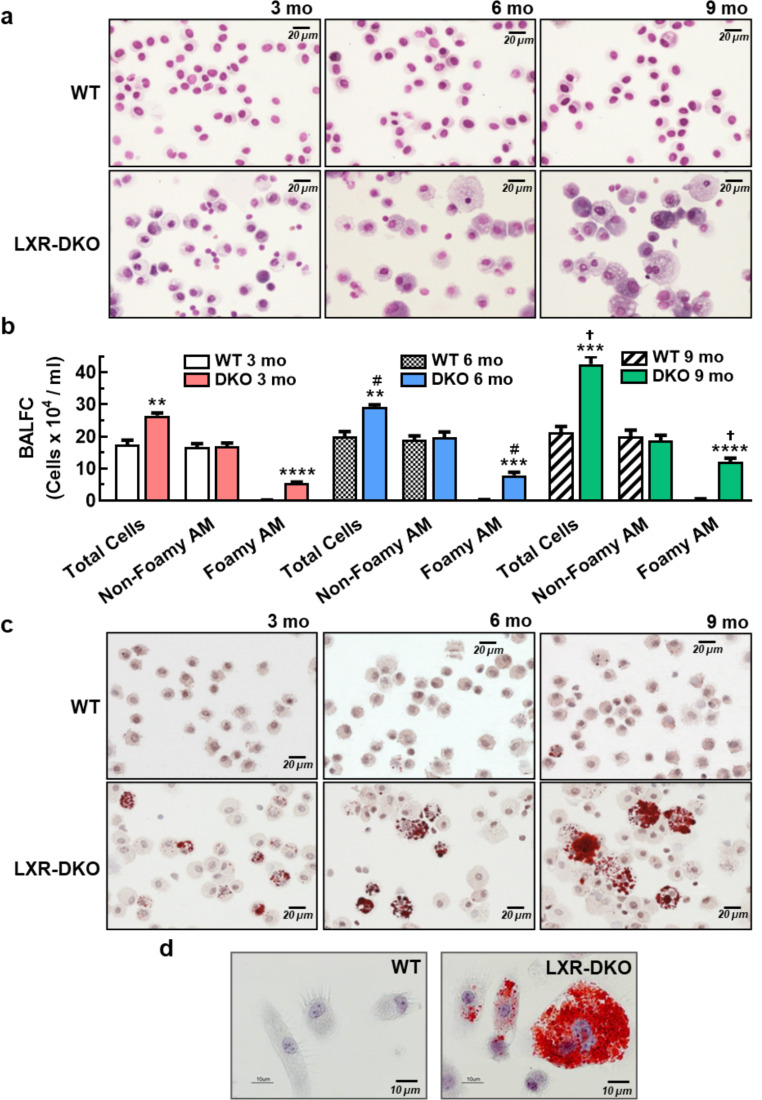
**Accumulation of lipid-laden AMs in LXR-deficient lungs**. **a**: Representative images (*n* = 5) of BAL cells, at 3- (left panels), 6- (middle panels) and 9-month-old (right panels) WT and LXR-DKO mice, stained with H&E. Scale bars, 20 μm. **b**: Total cells, non-foamy and foamy AM in the BAL of 3- to 9-month-old WT and LXR-DKO were counted by contrast-phase brightfield microscopy. Data represent the mean and standard error of three experiments (*n* = 4 per genotype). Unpaired Student’s t test was used for two-group comparisons; **p < 0.01; ***p < 0.001; ****p < 0.0001 compared to WT group; ^**#**^*p* < 0.05 compared to 3 months old and *P* < 0.05 compared to 6 months old mice. **c**: BAL cells from3- (left panels), 6- (middle panels) and 9-month-old (right panels) WT and LXR-DKO mice were subjected to Oil-Red-O staining and analyzed by contrast-phase brightfield microscopy. Scale bars, 20 μm. **d**, Representative images of BAL cells from 9-month-old WT and LXR-DKO mice (*n* = 4 per genotype) stained with Oil-Red-O (100x). Scale bars, 10 μm

Furthermore, taking into account the crucial role of the AM in the homeostasis of the surfactant material and given that approximately 90% of the material is constituted by phospholipids, we performed a first approach to analyze the intracellular phospholipid load of the AM. The simultaneous detection of steatosis and phospholipidosis in vitro (Fig. 2b) revealed that LXR-deficient AMs that had turned into foam cells and accumulated neutral lipids (bodipy, green fluorescence) in the form of vacuoles in their cytoplasm showed weaker labeling of intracellular phospholipids (phospholipidosis, red fluorescence). This suggests that cholesterol accumulation by AMs in the LXR-DKO lung may lead to disruption of their phospholipid metabolism. Moreover, ultrastructural analysis of LXR-DKO lungs by electron microscopy revealed a clear morphological alteration of both T2Ps and AMs. T2Ps appeared hypertrophic with aberrant lamellar bodies (containing the *de novo* surfactant material) and exhibited increased size and electrodensity compared to LXR-WT lungs (Fig. 2c). While mitochondria (orange arrowheads) and other organelles are similar in size in both genotypes, lamellar bodies (red arrowheads) are the only organelles that show increased size in LXR-DKO T2Ps. In addition, AMs of LXR-DKO mice appeared hypertrophied compared to those in WT lungs (Fig. 2c) and showed large intracytoplasmic inclusions whose thin elongated shape suggests cholesterol accumulation in the form of “cholesterol needles” [24]. Likewise, counting of foamy and non-foamy AMs from BAL showed a progressive increase in the number of both total cells and foamy AMs in BAL from 3- to 9-month-old LXR-DKO mice compared to BAL from WT mice (Fig. 3a, b). OR-O-positive AMs (Fig. 3c, d) from LXR-DKO BAL showed lipid accumulation in the macrophage cytoplasm, confirming that LXR deficiency leads to the development of lipidosis in AM.

To evaluate the origin of lipidosis, we analyzed the whole lung and BALF lipid profiles of LXR-DKO and WT mice by HPTLC. As shown in Table 1, the levels of esterified cholesterol -the biochemical storage form of cholesterol in the cell- were particularly elevated in both the lung and BALF of LXR-DKO mice, whereas triglyceride levels were significantly reduced in the lung of LXR-DKO mice. Besides neutral lipids, lipid analyses also showed a significant reduction in polar lipids, including sphingolipids and phospholipids (overall percentage of 19%). Thus, except phosphatidylcholine, all other phospholipids were significantly reduced in the lungs of LXR-DKO mice, revealing a massive dysregulation of surfactant lipid composition. Comparatively, changes in polar lipids were much less evident in BALF samples (average 4.9%), except phosphatidylglycerol, which was undetectable in BALF of LXR-DKO mice. Taken together, these results indicate that loss of LXR causes an alteration in the homeostasis of cholesterol and phospholipid components of surfactant material in the lung, which appear to affect both the ability of AMs to store cholesterol and of T2Ps to synthesize surfactant material via lamellar bodies.

**Table 1:**
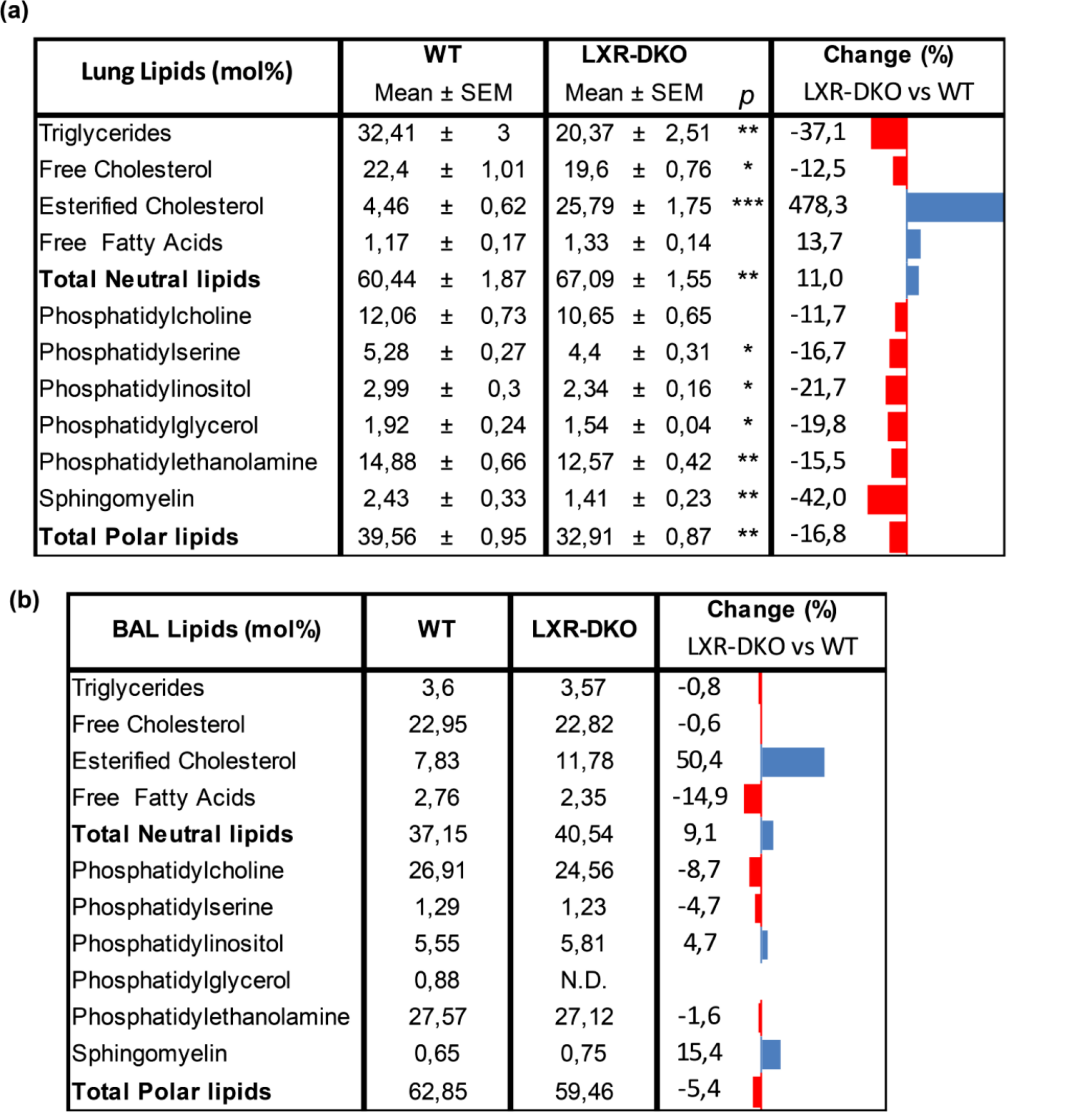
**Altered lipid content in the lungs and BALF of LXR-DKO mice**. Lipid levels were extracted and determined as described in material and methods. a, Lipid levels in WT or LXR-DKO lungs from 6-month-old mice. Data are expressed as mole percent (mol%) and represent the mean and standard error of mean (SEM). *N* = 5 (WT), *N* = 6 (LXR-DKO). Unpaired Student’s t test was used to analyze the statistical differences in lipid content between WT and LXR-DKO lungs. *p < 0.05; **p < 0.01; ***p < 0.001. b, Lipid levels in the BALF of 6-month-old WT or LXR-DKO mice. Data are expressed as mole percent (mol%) of pooled samples from *N* = 9 mice of each genotype

### LXR regulates the expression of genes important for pulmonary surfactant homeostasis

Given the crucial roles of LXRs in lipid and inflammatory homeostasis in several tissues, we next studied the transcriptional activity of LXR in lung tissue. To explore this, we activated LXR in vivo by intraperitoneal injection (10 mg/kg, for 3 days) of the LXR agonist, GW3965, and then analyzed the expression levels of established LXR target genes such as *Abca1*, *Abcg1* and *Srebf1* [25]. As shown Fig. S2, the transcript levels of these genes, analyzed by real-time RT-QPCR, were significantly induced in WT mice in response to GW3965, indicating that LXR activity is inducible in the lung by an acute administration of synthetic LXR agonist.

**Fig. 4:**
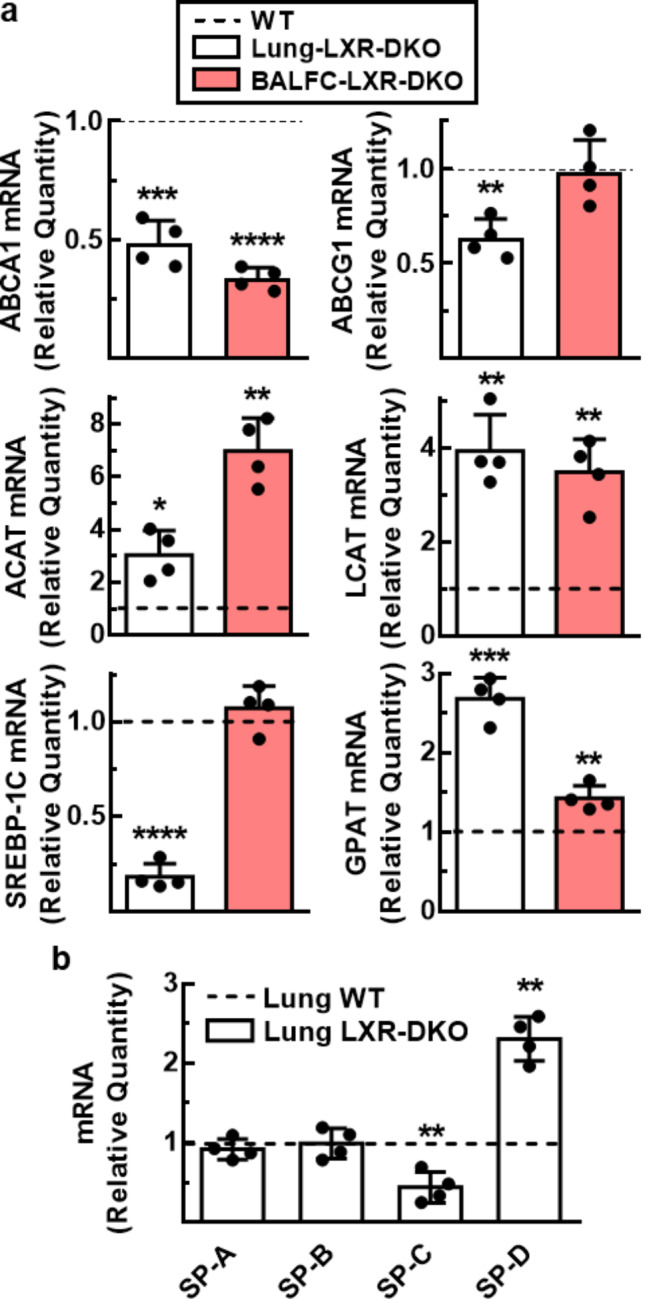
**Impaired pulmonary surfactant homeostasis gene expression in LXR-deficient mice**. Transcript levels of genes involved in lipid (**a**) and protein (**b**) metabolism of pulmonary surfactant from BAL cells (BALC) and lung tissue from WT and LXR-DKO 6-month-old mice were analyzed by real-time qPCR. WT gene expression values were normalized to 1 and used as control (dashed line). Data represent the mean and standard error of three experiments (*n* = 4 mice per genotype). Unpaired Student’s t test with Welch’s correction was used for two-group comparisons; *p < 0.05; **p < 0.01; ***p < 0.001; ****p < 0.0001 compared to WT group

Considering the central role of LXR in the maintenance of lipid homeostasis as cholesterol sensors, the expression of those LXR target genes whose dysregulation could be involved in the development of the LXR-DKO phenotype was analyzed, in whole lung tissue and BAL cells by real-time RT-QPCR. As shown in Fig. 4a, loss of LXR resulted in a large decrease in ABCA1 and ABCG1 mRNA levels in lung and ABCA1 in BAL cells, consistent with the cholesterol accumulation observed in LXR-deficient lung cells (AM and T2P) and BALF. Furthermore, the expression of ACAT1 (acetyl-CoA-acetyltransferase 1) and LCAT (lecithin cholesterol-transferase), enzymes involved in cholesterol esterification, was elevated in both lung and BAL cells of the LXR-DKO compared to WT. SREBP-1C-dependent lipogenic pathway was also reduced in lung LXR-DKO cells. In contrast, mRNA levels of glycerol-3-phosphate acyltransferase (GPAT), a limiting enzyme of the *de novo* pathway of glycerolipid synthesis that plays a key role in regulating triglyceride and phospholipid synthesis, were significantly increased in both lung tissue and BAL cells. Thus, loss of LXR causes the accumulation of cholesterol inside the cells and the derepression of enzymes necessary for the transformation of free cholesterol into esterified cholesterol. Overall, these results are consistent with the altered levels of cholesterol, triglycerides and phospholipid components of the surfactant material found in BALF and lung tissue from LXR-DKO mice.

Our results presented above showed that T2Ps displayed aberrant lamellar bodies in LXR deficient mice. Since these subcellular structures are the origin of extracellular surfactant fluid, we next analyzed by RT-qPCR the expression of surfactant component proteins in lung tissue from WT and LXR-DKO mice. Although SP-A and SP-B mRNA levels remain unchanged (Fig. 4b), SP-C and SP-D levels were significantly altered in LXR-DKO lung compared to WT lung, suggesting that SP-C and SP-D protein expression may be under the control of LXR activity and that the absence of LXR in the lung results in altered protein components of surfactant. Furthermore, since SP-C is synthesized mainly by T2Ps, these data suggest that this cell type is likely involved in the development of the LXR-DKO phenotype. Taken together, our results indicate that, in addition to altered expression of several enzymes that regulate cholesterol homeostasis and phospholipid synthesis in the lung, loss of LXR results in abnormal expression of surfactant component proteins, leading to de novo synthesis of defective surfactant material by T2Ps.

### Role of alveolar macrophages in lung histopathology in LXR DKO mice

**Fig. 5:**
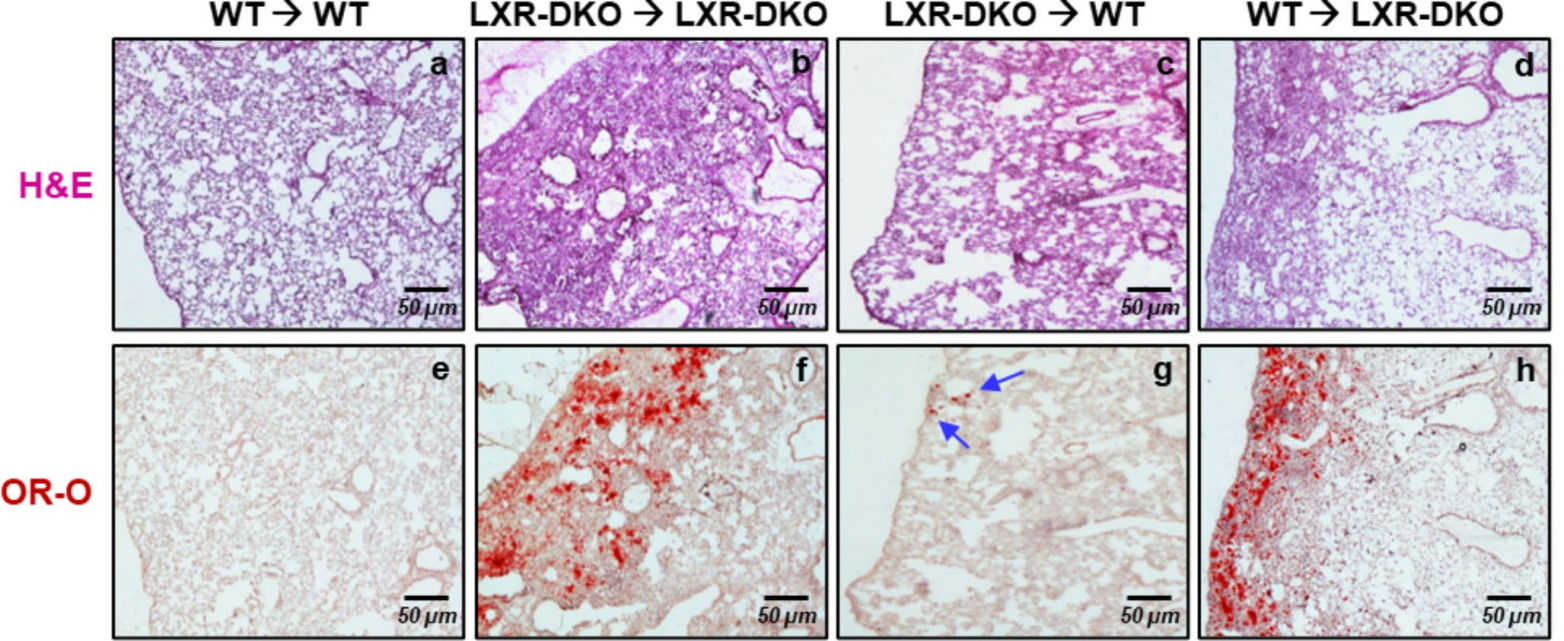
**Lung histopathology and lipid accumulation in WT and LXR-DKO mice after bone marrow transplant**. Morphology (H&E) and lipidosis (Oil-Red-O staining) were assessed in consecutive lung sections from wild-type and LXR-DKO mice 16 weeks after reciprocal bone marrow transplantation (labels: donor→recipient). Scale bars, 50 μm. Images are representative of two independent experiments with five to six mice per group

LXRα-specific transcriptional activity has been reported to be critical for macrophage development in the liver and spleen [20, 26]. Our results presented above have shown that deletion of both LXRα and LXRβ was required to develop an accumulation of foamy AMs in the lungs. To evaluate the contribution of macrophage LXR activity to the development of the lung phenotype of LXR-DKO mice, we performed bone marrow (BM) transplant (BMT) experiments to achieve AM replacement in the lung. First, we confirmed that 12 weeks post-transplant was an adequate time at which nearly all AM in recipient mice had been replaced by the donor BM by using BM progenitors from Csf1r-EGFP (MacGreen) transgenic mice, which contain a bone marrow-derived myeloid lineage expressing the green fluorescent protein (EGFP). Double immunofluorescent detection (Fig. S3a) in lung sections of mice transplanted with Csf1r-EGFP bone marrows revealed GFP signal co-localizing with the CD68 ^+^ macrophages, indicating successful replenishment of AMs in recipient mice. Next, we performed reciprocal BMTs using bone marrow from either WT or LXR-DKO mice and transferred into the indicated irradiated recipient mice. After 24 weeks post-transplant, consecutive lung sections were analyzed by histological techniques with hematoxylin-eosin and OR-O. Unexpectedly, transplant of bone marrow from wild-type donors did not ameliorate lipidosis and congestion in the subpleural zone of the lung of LXR-DKO recipient mice. As shown in Fig. 5, LXR-DKO recipient lungs showed infiltrates and oil-red positive cells within the subpleural area (Fig. 5d, h), similar to LXR-DKO control transplanted lungs (Fig. 5b, f). Consistent with this, a reciprocal transplant of BM from LXR-DKO donors resulted in minimal lipid accumulations in the subpleural zone (blue arrows) of the lung of WT mice (Fig. 5c, g). Finally, to rule out the possibility that the inability of WT transplanted macrophages to ameliorate the lipidosis present in LXR-deficient mice was due to unproductive replacement of the pre-existent foamy LXR-DKO AMs present in congested subpleural areas, we performed additional BMT experiments using Csf1r-EGFP donor BM transplanted into LXR-DKO mice. Analysis of GFP expression in consecutive lung sections confirmed the presence of GFP + macrophages in the subpleural area of the lung, thus indicating the overall efficacy of the transplantation. As shown in Fig. S3b, the subpleural area exhibited GFP signal in both WT and LXR-DKO mice. Moreover, this signal was coincident with that of oil red-positive cells (foamy AMs) in LXR-DKO recipient lungs (red dashed line), confirming that myeloid cells have been successfully replaced in the lung tissue. Altogether, these experiments indicated that, although macrophages participate in the pathogenesis of the observed phenotype, the contribution of LXR deficit in AMs cannot be considered exclusively as the main cause of the extensive lipidosis and inflammation observed in LXR-DKO mice.

### Deficient regulation of surfactant metabolism by type II pneumocytes in LXR-DKO mice

Our results so far suggested that tissue cells other than myeloid cells, such as surfactant fluid-producing T2Ps, may be responsible for lung histopathology in LXR DKO mice. First, we analyzed LXR activity in a murine cell model of T2Ps, using the MLE-12 pneumocyte cell line (CRL-2110™), to clarify whether LXR activity could be involved in the processing of pulmonary surfactant by T2Ps. Therefore, we examined the expression of LXRα and LXRβ in MLE-12 cells (Fig. S4) using peritoneal macrophages as a cell reference. As shown in Fig. S4a, MLE-12 cells expressed LXRα and LXRβ mRNA, although at lower levels than peritoneal macrophages. In addition, unlike peritoneal macrophages, MLE-12 cells showed similar levels of LXRα and LXRβ mRNA (Fig. S4a) and proteins (Fig. S4c). Subsequent analysis of the expression of ABCA1 in MLE-12 cells showed that its mRNA (Fig. S4b) and protein (Fig. S4c) levels increased in response to GW3965, whereas the mRNA levels decreased dramatically when the cells were co-treated with the antagonist GW233 (Fig. S4b). Analysis of pro-SP-C protein expression (Fig. S4c) by western-blot in MLE-12 cells confirmed expression of the surfactant protein and its levels did not change in response to GW3965. These results indicate that both LXRs are present and active in the MLE-12 pneumocytes, which expresses the pro-SP-C surfactant protein in culture and therefore represents a suitable model for further analysis.

**Fig. 6:**
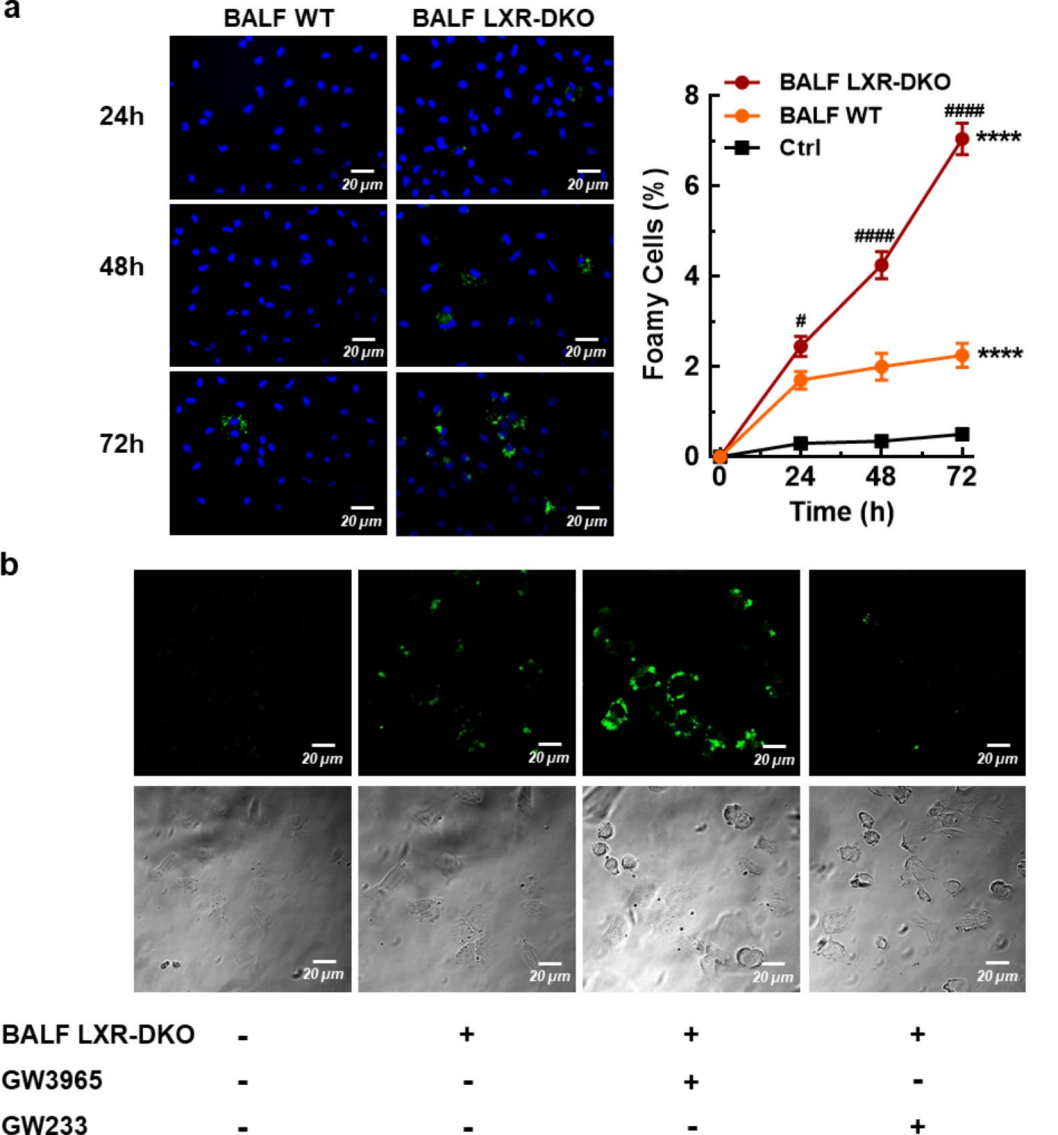
**Modulation of LXR activity affects the handling of surfactant material by MLE12 cells**. In vitro lipidosis model: Peritoneal macrophages from WT mice were cultured and exposed to BALF from WT and LXR-DKO mice. Subsequently, (**a**) fluorescence microscopy analysis and percentage of foam cells were determined using Bodipy 493/503 after 24, 48 and 72 h incubation of peritoneal macrophages with BALF. A representative image is shown (left panel) and the data are presented as the mean and standard error (right panel), *n* = 4 mice per genotype. Kruskal-Wallis with a post hoc Dunn’s test was used for multiple comparisons; ****p < 0.0001 and **p < 0.001 compared to control group; ^***#***^*P* < 0.05 and ^***##***^*P* < 0.001 compared to BALF WT groups. (**b**) MLE-12 cells were pre-incubated with GW3965 agonist or GW233 antagonist for 24 h and then exposed to LXR-DKO BALF for additional 24 h. Lipid uptake was measured by bodipy 493/503 signal (A-G) or bright field (B-H) using confocal microscopy. Cells not exposed to BALF were used as control. A representative image of 3 independent experiments is shown. Scale bars of all images are 50 μm

Subsequently, we modeled in vitro the environmental conditions of lipidosis in which LXR-DKO lung pneumocytes are found by adding bronchoalveolar fluid to cultured cells. Initially, the effect of BALF obtained from WT and LXR-DKO lungs on WT peritoneal macrophage cultures was analyzed. The percentage of foamy cells was determined by using Bodipy 493/503, after 24, 48 and 72 h incubation of macrophages with fractions of BALF. As shown Fig. 6a, the presence of WT BALF resulted in low lipid accumulation in macrophages at 72 h. Importantly, the magnitude and timing of foamy cell formation was robustly observed earlier when macrophages were cultured in the presence of LXR-DKO BALF (Fig. 6a), emphasizing the lipid accumulation inside the macrophages observed from 24 h to 72 h of incubation with BALF LXR-DKO. These results indicate that bronchoalveolar fluid from LXR-DKO animals is capable of inducing macrophage-lipidosis in culture. We then hypothesized that we could translate this model to drive lipid handling in vitro in T2Ps, cells with the capacity to uptake and recycle surfactant material. Thus, we cultured MLE-12 cells with LXR-DKO BALF in the presence of GW3965 agonist or G233 GW233 antagonist and measured lipid uptake by analyzing bodipy 493/503 signal. As shown in Fig. 6b, pneumocytes cultured with BALF experienced a slight increase in lipid accumulation in their cytoplasms, as did peritoneal macrophages, confirming the validity of this model with MLE-12 cells. Interestingly, lipid uptake was strongly enhanced in the presence of GW3965 agonist, an effect that was completely reversed when MLE-12 cells were co-treated with GW233 antagonist (Fig. 6b). These data indicate that LXR activation is important for the uptake and handling of surfactant material by T2Ps expressing LXRs. Data also suggests that diminished LXR activity could lead to defective surfactant handling by T2Ps and, consequently, accumulation of this material in the alveolar space.

**Fig. 7:**
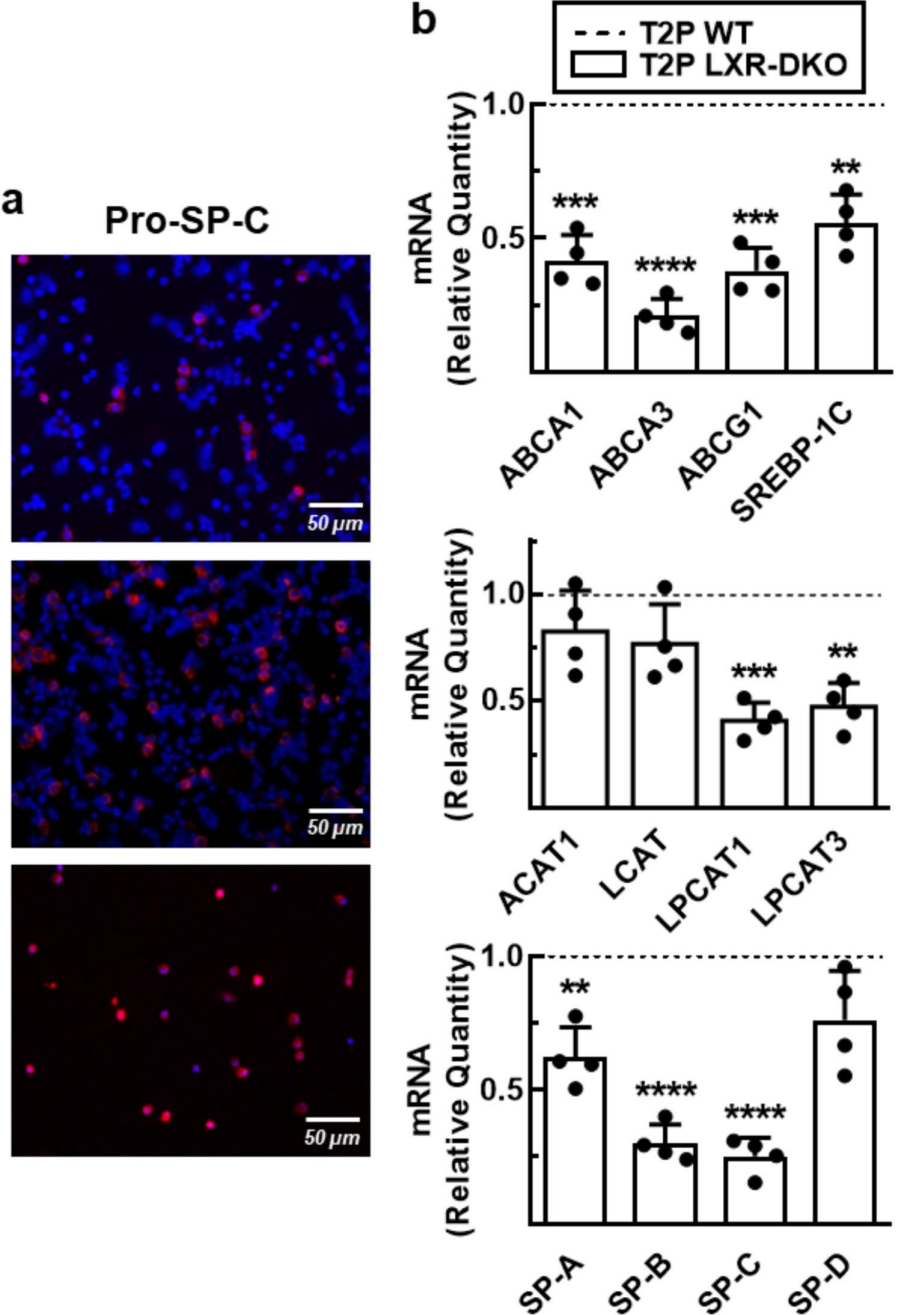
**LXR deficiency alters primary type 2 pneumocytes gene expression**. **a**, T2Ps were isolated from WT and LXR-DKO 6-month-old mice by enzymatic digestion and immunomagnetic purification, and subsequently cultured on a Matrigel-coated surface. Enrichment of T2P through the isolation method was monitored by pro-SPC expression by immunocytochemistry in the total lung homogenate (upper panel), after recovery of non-adherent cells (middle panel) and at the end point of isolation after the positive selection column (low panel). A representative image (*n* = 4) is shown. Scale bars of all images are 50 μm. **b**, Transcript levels of genes involved in lipid (upper and middle panels) and protein (low panel) metabolism of pulmonary surfactant from T2P, isolated from WT and LXR-DKO 6-month-old mice, were analyzed by real-time qPCR. WT gene expression values were normalized to 1 and used as control (dashed line). Data represent the mean and standard error of three experiments (*n* = 4 mice per genotype). Unpaired Student’s t test with Welch’s correction was used for two-group comparisons; **p < 0.01; ***p < 0.001; ****p < 0.0001 compared to the WT T2P group

The results obtained with MLE-12 lung epithelial cells led us to study the role of LXRs in the regulation of surfactant material by primary T2Ps. These cells were isolated from mice using enzymatic digestion, immunomagnetic purification and cultured on a matrigel-coated surface. Monitoring of pro-SPC expression by immunocytochemistry revealed an enrichment of the T2Ps population by 80–95% (Fig. 7a) during routine isolation of these cells from murine lungs. Furthermore, RT-qPCR analysis of the expression of LXR target genes (Fig. 7b, upper panel) such as *Abca1*, *Abcg1* and *Srebf1* in primary pneumocytes isolated from LXR-DKO mice showed significantly reduced mRNA levels compared to WT mice, confirming that LXR is relevant for the expression of these genes in primary cells. Next, we analyzed other genes involved in lipid and protein synthesis related to the production and recycling of surfactant material by T2Ps. While ACAT1 and LCAT mRNA levels did not change, importantly, ABCA3 (membrane transporter of different classes of lipids associated with lamellar bodies) and LPCAT1/3 (lysophosphatidyl acyltransferases involved in the recycling or remodeling pathway, Land’s cycle, of phospholipids) mRNAs were drastically diminished in T2Ps from LXR-DKO mice compared to the WT (Fig. 7b, upper and middle panel). Furthermore, the expression levels of the surfactant component proteins, SP-A, but especially SP-B and SP-C, were significantly decreased in the LXR-deficient T2P (Fig. 7b, lower panel). Taken together, these results demonstrate that LXR is present in primary T2PS and its activity is crucial for the correct synthesis and recycling of surfactant material, and suggest that defective LXR transcriptional activity by the T2P could be the main cause of the lung histopathology found in LXR-DKO mice.

### Inflammation and impaired pulmonary function in LXR DKO lungs

**Fig. 8:**
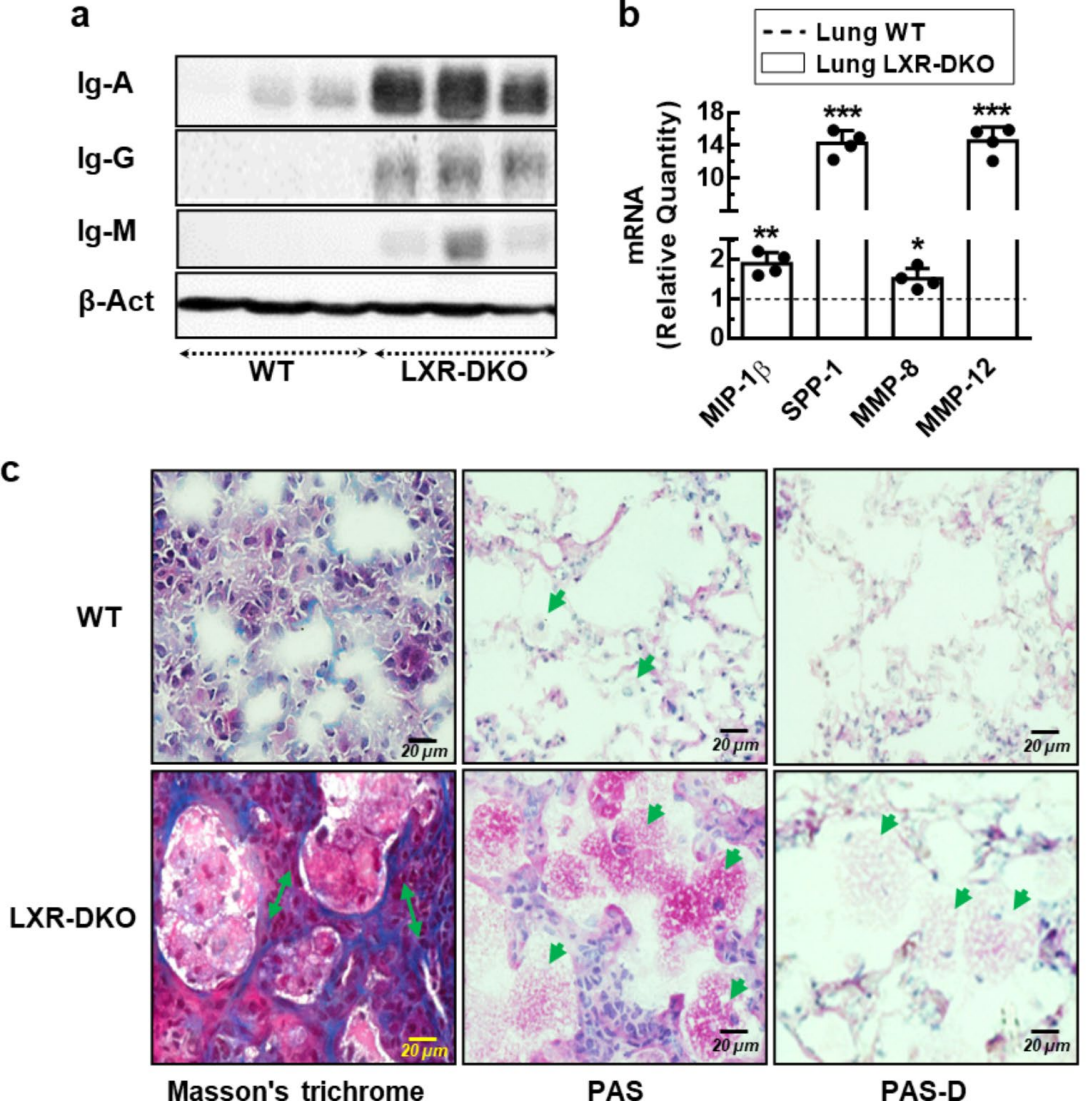
**LXR inactivation leads to a chronic inflammatory process in the lung**. **a**, Whole cell lysates were prepared from homogenized lung tissue cells from perfused WT and LXR-DKO 6-month-old mice and IgA, IgM or IgG proteins were analyzed by Western blot. Membranes were stripped and reprobed with β−actin antibody as loading control. A representative western blot (*n* = 3 mice) is shown. **b**, Transcript levels of inflammatory related-genes, from homogenized lung tissue cells from WT and LXR-DKO 6-month-old mice, were analyzed by real-time qPCR. WT gene expression values were normalized to 1 and used as control (dashed line). Data represent the mean and standard error of three experiments (*n* = 3 mice per genotype). Unpaired Student’s t test with Welch’s correction was used for two-group comparisons; *p < 0.05; **p < 0.01; ***p < 0.001 compared to WT group. **c**, Masson’s trichrome (MT) staining (left panels) and periodic acid-Schiff (PAS) staining (middle panels) were evaluated in consecutive lung sections from 6-month-old WT and LXR-DKO mice. Pretreatment of tissues with PAS-diastase (α-salivary amylase) (PAS-D) (right panels) was assessed to confirm PAS-positive material. A representative image of 4 mice per genotype is shown. Scale bars of all images are 20 μm

The alteration of lung morphology in LXR-deficient mice, characterized by the progressive development of lipidosis and infiltration of immunocompetent cells, are pathological events associated with an inflammatory process that could affect lung function. The development of the inflammatory process was confirmed by the subsequent detection of elevated levels of immunoglobulins IgA, IgG and IgM in perfused LXR-DKO lung tissue (Fig. 8a). These data indicate that the numerous leukocyte infiltrates, previously observed in the lung tissue of LXR-DKOs, may be producing these immunoglobulins and contributing to the proinflammatory environment. Furthermore, the expression of genes whose overexpression is related to the development of an inflammatory process: *Spp1* (SPP-1, secreted phosphoprotein-1), *Mip-1β* (MIP-1β, macrophage inflammatory protein-1β), *Mmp-8* and *Mmp-12* (MMP-8 and MMP-12, matrix metalloproteinase-8 and − 12), was analyzed in WT and LXR-DKO lungs. As shown in Fig. 8b, MMP-12, SPP-1 and MIP-1β expression levels were increased, in LXR-DKO lungs. These metalloproteinases have been shown to promote the recruitment of macrophages and other immune cells to sites of inflammation and fibrosis [27, 28].

To evaluate if this inflammation also results in fibrosis in the lungs of LXR-DKO, we performed Masson’s trichrome staining of lung sections. As shown in Fig. 8c, collagen fibers (blue color), in WT lungs, were present forming part of a thin connective tissue layer accompanied by laxly arranged fibroblasts (nuclei, purple color), which constitute the alveolar septa, separating and supporting the alveolar sacs. In contrast, the stroma constituting the alveolar septa appeared congested (Fig. 8c, double green arrows) in LXR-DKO lungs, constituted by increased cellularity and thick collagen fibers that patently occupy almost the entirety of the alveolar septa. Moreover, the alveolar space was enlarged in LXR-DKO mice due to a nearly complete occupied space by numerous AMs (nuclei, purple color, cytoplasm, red color), which were foamy and hypertrophied. Next, we analyzed by periodic staining with Schiff acid (PAS) the possible accumulation of glycoprotein components of the surfactant material in the alveolar space because of an alteration in its synthesis and recycling in LXR-DKO lungs. As shown in Fig. 8c, staining detected PAS-positive material filling the alveolar spaces of LXR-DKO lungs (pink color) compared to WT, corresponding to accumulated surfactant material. In addition, this staining revealed PAS-positive material in the cytoplasm of AMs (Fig. 8c, green arrowhead), coincident with the OR-O-positive foamy macrophages observed previously. Pretreatment of tissues with PAS-D diastase (α-salivary amylase) confirmed that it is indeed a PAS-positive material (Fig. 8c). Taken together, these results suggest that inactivation of LXR leads to a chronic inflammatory process in the lung as a consequence of mismanagement of surfactant biosynthesis, ultimately resulting in progressive alveolar proteinosis aggravated with age.

**Fig. 9:**
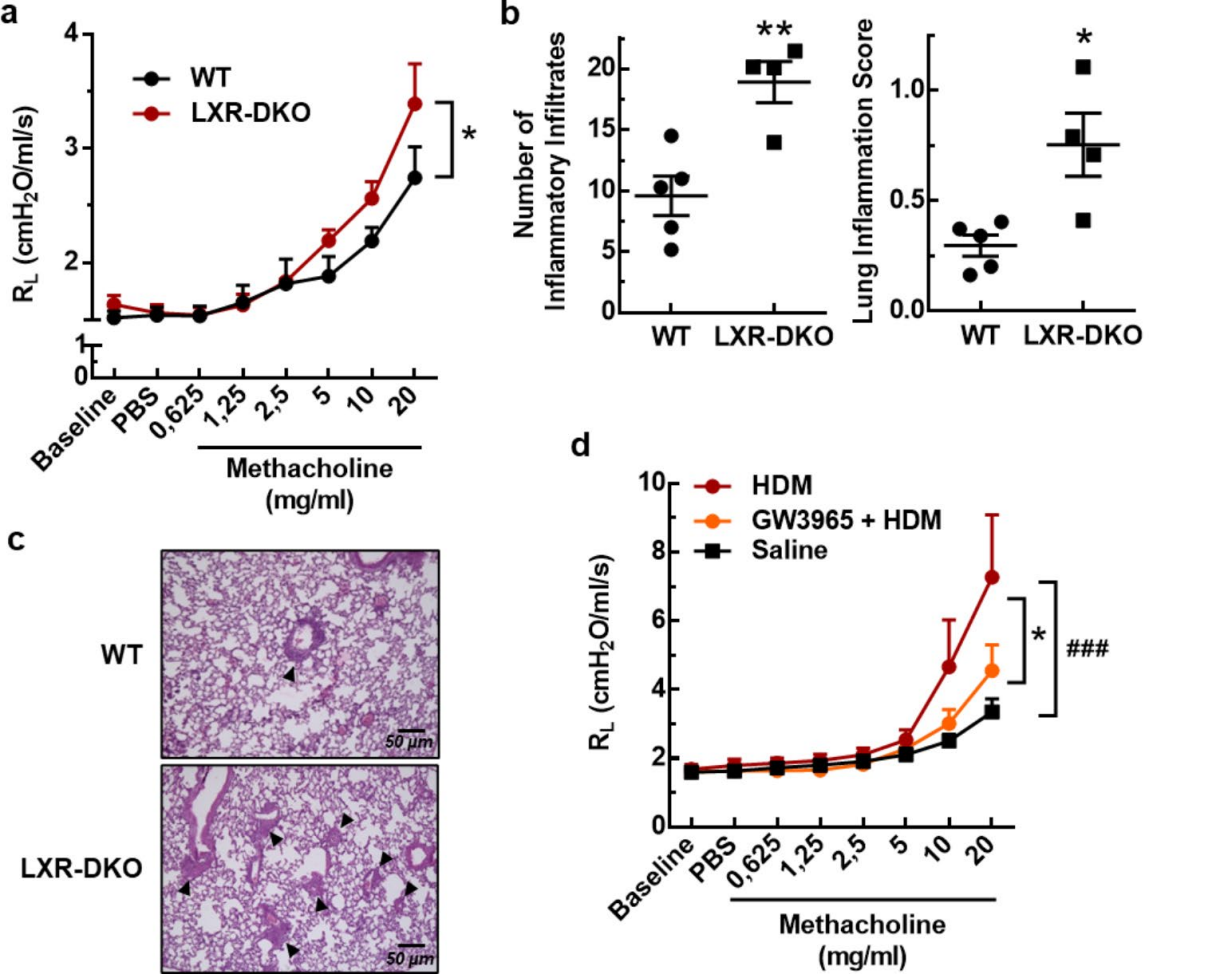
**LXR pharmacological activation ameliorates lung reactivity in mice exposed to HDM**. WT or LXR-DKO 3-month-old mice were administered with HDM extract daily intranasally at a dose of 25 µg/mouse for 10 consecutive days. **a**, Lung resistance (RL) to increasing doses of methacholine was assessed 24 h after the last HDM exposure. Data represent the mean and standard error of *n* = 5 (WT) or *n* = 4 (LXR-DKO) mice. The effect of genotype was analyzed using one-way ANOVA with a post hoc Bonferroni test; * *p* < 0.05. **b**, Histological analysis of the number (left panel) of perivascular and/or peribronchiolar inflammatory infiltrates in the lung. The lung inflammation score (right panel) was calculated as described in Methods. Unpaired Student’s t test; *p < 0.05, **p < 0.01 compared to WT group. **c**, Representative images from the lungs of HDM-exposed WT or LXR-DKO mice. **d**, 8- week- old WT mice were separated in three groups: non-sensitized mice (Saline group, *N* = 11) received daily an intranasal administration of physiological saline and an intraperitoneal administration of vehicle (DMSO in PBS); HDM-sensitized mice received daily an intranasal administration of HDM and an intraperitoneal administration of either vehicle (DMSO in PBS) (HDM group, *N* = 10) or of the LXR agonist GW3965 (20 mg/kg) (GW3965 + HDM group, *N* = 11). Administration of the LXR agonist or vehicle was initiated one day before the start of sensitization. Lung resistance (RL) to increasing doses of methacholine was assessed 24 h after the last HDM exposure. Pooled data from two independent experiments. The effect of treatment was analyzed using one-way ANOVA with a post hoc Bonferroni test. Mean ± SEM; ^***###***^*P* < 0.0001 compared to saline group; *p < 0.01 compared to HDM group

Finally, we used a mouse model of house dust mite (HDM)-induced allergic airway inflammation to analyze whether the baseline lipid and immune defects developed by LXR-DKO mice could affect HDM-challenged lung functions [29]. To this purpose, WT and LXR-DKO mice were sensitized through intranasal instillations of an HDM extract for 10 days and then the airway reactivity to a methacholine challenge and the lymphocytic infiltration in the lungs were analyzed. HDM-exposed LXR-DKO mice presented more pronounced airway resistance in response to methacholine challenge (Fig. 9a) and increased infiltration in perivascular and peribronchiolar areas (Fig. 9b, c), compared with HDM-exposed controls. Next, WT mice were stimulated with an LXR agonist, before HDM exposure and during the entire course of sensitization. Pretreatment with GW3965 markedly improved the lung response to methacholine challenge in WT mice exposed to HDM (Fig. 9d). These results indicate that LXR pharmacological activation might be useful as a therapeutic alternative in pulmonary allergic inflammatory diseases. Taken together, our results demonstrate that LXR is involved in the control of surfactant synthesis and recycling by T2Ps and AMs, and that LXR deficiency promotes a chronic inflammatory process that impairs lung function.

## Discussion

LXRs are transcription factors that regulate crucial processes in lipid metabolism and also exert important functions in inflammation and host immunity [30, 31]. One of the most important mechanisms of mammalian innate immunity resides in the respiratory tract, where AMs, T1Ps and T2Ps form an important barrier against the entry of pathogens [5]. In this study, we used mice deficient in LXRα and LXRβ to investigate the role of the LXR signalling pathway in lung histophysiology and, subsequently, the underlying mechanism of the inflammatory process caused by their loss in the lung. The experimental results presented clearly demonstrate that LXRs in the lung have an essential role in maintaining lung surfactant homeostasis and that their defect leads to a pathological pulmonary process with inflammatory characteristics.

Architecture and physiology of the lungs were severely affected by the loss of LXR activity, resulting in the appearance of lipid accumulation, fibrosis and tissue congestion, characterized by an abundant cellular infiltrate of B and T lymphocytes in the subpleural zone. Similar features are in part observed in knockout animals for some key LXR target genes, such as *Abca1* -/- [32] or *Abcg1*-/- [24], including the development of lung lipidosis, supporting that LXR activity is key to modulate lipid metabolism in the lung. In addition, the altered lung areas become more prominent with age and occupy a larger proportion of the lung parenchyma in LXR-DKO mice, but this is not observed in single *Lxr*α-/- and *Lxrβ*-/- animals, indicating that both LXR isoforms share partially overlapping functions in lung physiology and that double deficiency is necessary to develop the pathological lung architecture. Although the development of morphologic changes in the lungs of LXR-DKO mice is evident from 3 months of age, it is plausible that the LXR-DKO mice may accumulate some foamy macrophages before 3 months of age.

The changes observed in LXR-DKO lungs animals included increased numbers of foamy AMs in both the parenchyma and the BAL. These AMs exhibit hypertrophic morphology and large intracytoplasmic inclusions resembling “cholesterol needles” [24]. Defective cholesterol efflux by LXR-DKO AMs, similar to *Abca1* -/- and *Abcg1*-/- mice, explains the high number of foamy cells. In fact, gene expression analysis showed that LXR deficiency results in decreased expression of ABCA1 and ABCG1, leading to cholesterol accumulation in the lung and BALF. In addition, the enrichment of esterified cholesterol shown by analysis of BALF and lung in LXR-DKO mice could be the result of overwhelmed foamy macrophages not being able to take up excess cholesterol from the extracellular medium, a function that is crucial for the role of AM in the homeostasis of surfactant material [7]. The accumulation of cholesterol by LXR-deficient foamy AMs could interfere with their phospholipid metabolism, indicating that this phenotype probably reflects a deficit in their potential to recycle pulmonary surfactant.

Additional gene expression assays showed that LXR deficiency derepresses the expression of enzymes necessary for the conversion of free cholesterol into esterified cholesterol for efficient storage, thereby preventing the toxicity of excess free cholesterol. We also reason that, to counteract the reduced activity of the SREBP-1 C-dependent lipogenic pathway, the mRNA levels of GPAT, a limiting enzyme of the *de novo* pathway of glycerolipid synthesis that plays a key role in regulating triglyceride and phospholipid synthesis, were significantly increased in both lung tissue and BAL cells. These data are consistent with the higher levels of esterified cholesterol and lower levels of triglycerides found in BALF and lung tissue from LXR-DKO mice and suggest that accumulation of foamy AMs is a consequence of both a defect in the efflux of intracytoplasmatic cholesterol and high levels of cholesterol present in the extracellular surfactant material.

It is widely described that the lipid fraction of lung surfactant is composed mainly of phospholipids, and to a lesser extent of cholesterol [33]. Lower levels of phospholipids in both LXR-DKO lung and BALF and weaker labeling of accumulated phospholipids by AMs in LXR-DKO BALF indicate that LXR plays an important role in phospholipid metabolism necessary to produce surfactant material. Since T2P is the cell responsible for the *de novo* synthesis of pulmonary surfactant, the discovery of aberrant lamellar bodies in T2P cells of LXR deficient mice proves that LXR is crucial for the generation of proper surfactant material by T2P cells. In line with this, expression of surfactant proteins SP-C and SP-D were significantly decreased in LXR-DKO lung compared to WT lung, indicating malfunctioning of T2Ps. These results indicate loss of LXR results in abnormal expression of surfactant component proteins, leading to de novo synthesis of defective surfactant material by T2Ps.

Although LXR transcriptional activity has been reported to be important for macrophage development in the liver and spleen [20, 26], our results derived from bone marrow transplantation experiments, in which the AMs population in LXR-DKO lungs was effectively replaced by wild-type AMs, showed minimal resolution of the lung pathology. These observations suggest that, although AMs are altered and contribute to the phenotype of the LXR-DKO lung, the defects in this cell type are unlikely to be the triggering factor of the pathological phenotype. Thereby, LXR deficit in T2P cells is most likely a main driver of the extensive lipidosis and inflammation observed in LXR-DKO mice. In fact, pharmacological activation of the LXR pathway in MLE-12 pneumocytes along with incubation with LXR-DKO BALF resulted in enhanced lipid uptake, which was reverted by an LXR antagonist. These observations suggest that LXR activation is important for the uptake and handling of surfactant material by type II pneumocytes and LXR deficiency could therefore lead to accumulation of this material in the alveolar space.

ABCA1, ABCG1 and ABCA3 are present in the mammalian lung and are important for the proper transport of phospholipids and cholesterol from T2P and AM cells [34]. As expected, deficiency of LXR reduced the expression of classic LXR target ABCA1, ABCG1 and SREBP-1 C in T2P cells. It was indeed reported that both *Abca1* -/- and *Abcg1*-/- mice accumulate lipids into T2P cells [32] exhibit an age-related progressive pulmonary disease, including lipidosis and chronic inflammation [22]. Defects in cholesterol efflux gene expression in our LXR-DKO murine model are therefore consistent with nonfunctional surfactant recycling.

In addition to ABCA1 and ABCG1, the member of the ABC family that is more severely reduced in LXR-DKO T2P is ABCA3. This membrane transporter is found at high levels in T2P cells, specifically associated with the limiting membrane of lamellar bodies [35, 36]. The function of this transporter is essential for lamellar body formation and surfactant release into the airspaces [37, 38]. *Abca3* mutations are responsible for fatal surfactant deficiency and interstitial lung disease. Indeed, mutations in the human *Abca3* gene are associated with lethal respiratory distress syndrome in human newborns who die at birth because they do not contain adequate amounts of surfactant material [39]. Thus, reduced ABCA3 expression in LXR-DKO lungs explains in part the defective lamellar bodies observed in T2Ps in these mice.

LPCAT1 protein, the most abundant LPCAT protein family in alveolar type II cells, is responsible for the biosynthesis of the surfactant DPPC [10] and appears to be involved in the transport of this lipid from the ER to the cytoplasmic lamellar body for storage prior to secretion by T2P cells [40]. LPCAT1 expression has been shown primarily in the endoplasmic reticulum of lung alveolar T2P cells [41] whereas LPCAT3 basal expression has been described in lung tissue and identified as an LXR target gene [42, 43]. In both cases, the expression of these lysophosphatidyl acyltransferases in T2P of LXR-DKO lungs is reduced by more than 50% compared to the WT animal. This implies a deficit in the capacity of phospholipid recycling by LXR-DKO T2P and is consistent with the reduction in the levels of all phospholipid classes (in the case of PG the reduction is extraordinary) in the surfactant material of LXR-DKO mice. The reduced expression of LPCAT3 may also be a trigger for the inflammation observed in LXR-DKO lungs, as the LXR-LPCAT3 pathway has been identified as an important modulator of phospholipid metabolism, metabolic stress responses and inflammation in the liver [42]. On the other hand, decreased mRNA levels of the surfactant component proteins, SPs, in the LXR-deficient T2Ps indicate that the expression of these proteins is controlled by LXRs. Taking into account the critical role of these proteins in the proper synthesis of surfactant material and in innate immunity [5], these results suggest that SPs are important key players in the regulation of surfactant homeostasis and in the LXR-driven modulation of lung inflammation [44]. Although we cannot exclude the possibility that the immune activation/inflammation observed in our model in the lungs of LXR DKO mice could be fueled by an additional or independent process to the lipidosis, we have confidence to conclude that the deficit in surfactant homeostasis leads to an exacerbated inflammatory component in our model.

In this context of pulmonary lipidosis and inflammation, elevated levels of immunoglobulins IgA, IgG and IgM and raised expression of inflammation-related genes, such as *Spp-1*, *Mip-1β*, *Mmp-8* and *Mmp-12* in LXR-DKO lungs are hallmarks of an extensive inflammatory process. Several lung pathologies, including asthma, respiratory distress syndrome, and chronic obstructive pulmonary disease (COPD), have been associated with alterations in the regulation of metalloproteinases in lung tissue [45]. Moreover, the BALF analyzed from LXR-DKO lungs exhibited large hexagonal extracellular crystals, described as eosinophilic crystals [46, 47], which have been observed in other animal models that also exhibit pulmonary lipidosis, inflammation and fibrosis, such as *Abcg1* -/- mice [32]. The biochemical nature of these eosinophilic crystals could correspond to the formation of an extracellular complex of several proteins [22, 23]. The presence of these crystals in the lungs is often associated with asthma and pulmonary inflammation [48, 49].

Tissue repair is a physiological process that is triggered after initial tissue inflammation and involves the recruitment, activation, apoptosis, and eventual clearance of key effector cells. When the inflammation becomes chronic, fibrosis appears as a dysregulated repair process that is aberrantly and continuously “turned on” [50]. Consistent with our results, lipidosis and inflammation lead to pulmonary fibrosis in LXR-DXO mice. The lungs show highly congested alveolar septa, increased cellularity, and thick collagen fibers that appear to occupy almost the entire alveolar septa. Moreover, the alveolar space in LXR-DKO mice is severely altered due to an almost complete occupation by numerous hypertrophic and foamy AMs. These are the phenotypic hallmarks of pulmonary fibrosis, a chronic, progressive and fibrotic lung disease in which healthy tissue is replaced by an altered extracellular matrix and alveolar architecture is destroyed, severely compromising pulmonary physiology [51].

On the other hand, LXR-DKO lungs accumulate surfactant material in the alveolar space, a phenotypic hallmark of pulmonary alveolar proteinosis (PAP), because of the mismanagement of surfactant biosynthesis by T2P cells [6]. These results may indicate that the clearance and metabolism of surfactant material, mainly the phospholipid fraction, by AMs is impaired by the accumulation of neutral lipids in the cytoplasm due to a deficient efflux of cholesterol. Consequently, this could lead to an extracellular accumulation of surfactant material, which together with a pathological alteration of T2P, would result in impaired recycling of surfactant material from the alveolar space of LXR-DKO lungs. In fact, a positive periodic acid-Schiff (PAS) reaction in BAL indicates that extracellular protein material is present in PAP patients [52]. This pathology, sometimes associated with pulmonary fibrosis, involves impaired gas exchange that can lead to dyspnea, hypoxemia, or even respiratory failure and death [53]. In this regard, the pathological histology and biochemistry of LXR-DKO lungs is consistent with an increased airway reactivity and higher lung infiltration of inflammatory cells in a mouse model of allergic asthma [54, 55]. Treatment with an LXR agonist before and during sensitization to aeroallergens ameliorated airway resistance in WT mice, confirming that LXR play an important role in lung physiology and suggesting that agonistic pharmacology could be used to treat inflammatory lung diseases.

The experimental results presented here clearly demonstrate that in the lung, LXRs act as master transcription factors that regulate the expression of lipid metabolism-related genes in T2Ps and AMs and specifically of surfactant proteins in T2Ps. Consequently, inactivation of LXRs causes pulmonary lipidosis, chronic inflammation, fibrosis and proteinosis through defective synthesis *de novo* and recycling of surfactant material by T2Ps and defective uptake and degradation of excess surfactant by AMs, which are also incompetent in the efflux of excess cholesterol. We also present evidence that inadequate handling of surfactant by LXR-DKO lung impairs lung physiology and suggest that modulation of LXR nuclear receptors activity could be exploited as a pharmacological target to ameliorate inflammatory diseases of the lung.

### Electronic supplementary material

Below is the link to the electronic supplementary material.

Supplementary Material 1

## Data Availability

All data supporting the findings from this study are available from the corresponding author upon reasonable request.
