# Supplementary material for: Endogenous LXR signaling controls pulmonary surfactant homeostasis and prevents lung inflammation
Source: Cell Mol Life Sci. 2024 Jul 6;81(1):287. doi: 10.1007/s00018-024-05310-3 (PMC11335212; doi:10.1007/s00018-024-05310-3)
Supplement: Supplementary file 1 — Supplementary Material 1 [file 18_2024_5310_MOESM1_ESM.docx]

**
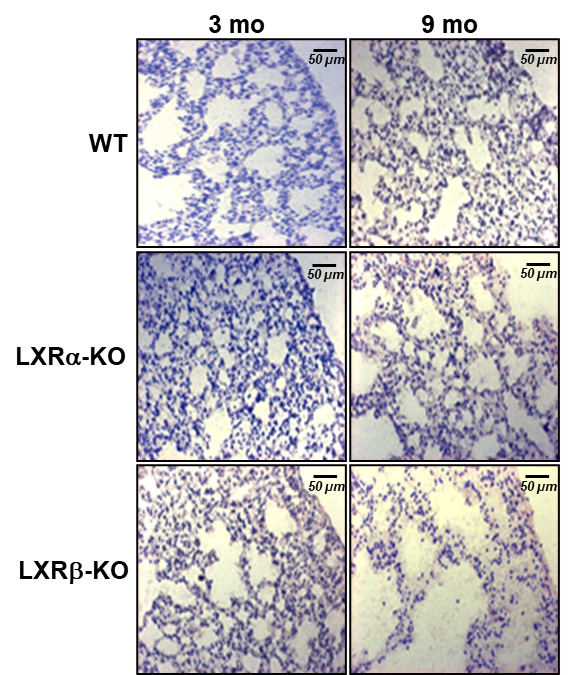
**

**Fig S1. Absence of morphological impairment in lungs from single LXR-KO isoforms.** WT, LXRα-KO and LXRβ-KO lungs from 3 and 9-month-old mice were subjected to macroscopic analysis by OR-O staining. Scale bars of all images are 50 μm. A representative image (n = 5) is shown.

**Fig S2. Analysis of LXR target gene expression in lung tissue**. Expression of *Abca1*, *Abcg1* and *Srebf1* was analyzed by real-time qPCR in lung tissue after intraperitoneal injection of PBS (control vehicle) or GW3965 in 3-month-old WT animals. Data represent the mean and standard error of three experiments (n = 4 mice). Control gene expression values were normalized to Ctrl = 1. Unpaired Student’s t test with Welch's correction was used for two-group comparisons; *****p < 0.05; ******p < 0.01 compared to Ctrl group.


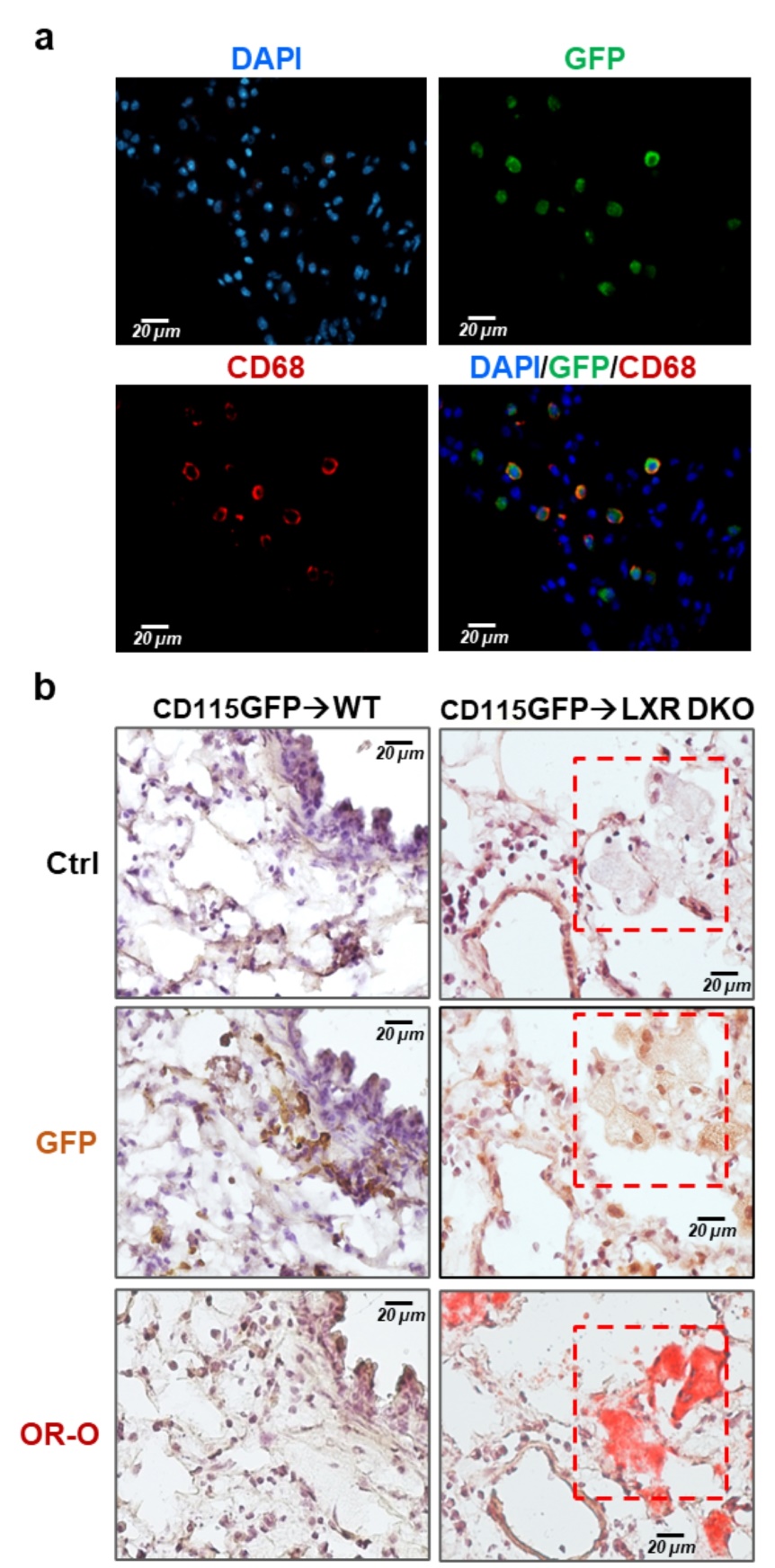


**Fig S3. Successful replenishment of AMs in recipient mice. a.** Analysis of GFP and CD68 expression were assessed in consecutive lung sections from mice 24 weeks after reciprocal bone marrow transplantation (CD115-GFP → LXR-DKO). Images are representative of two independent experiments with five to six mice per group. Scale bars, 20 μm. **b.** Analysis of GFP expression and lipidosis (Oil-Red-O staining) were assessed in consecutive lung sections from wild-type and LXR-DKO mice 24 weeks after reciprocal bone marrow transplantation (labels: donor→recipient). Images are representative of two independent experiments with six-eight mice per group. Areas marked with red dashed line indicate coinciding areas in consecutive sections Scale bars, 20 μm.


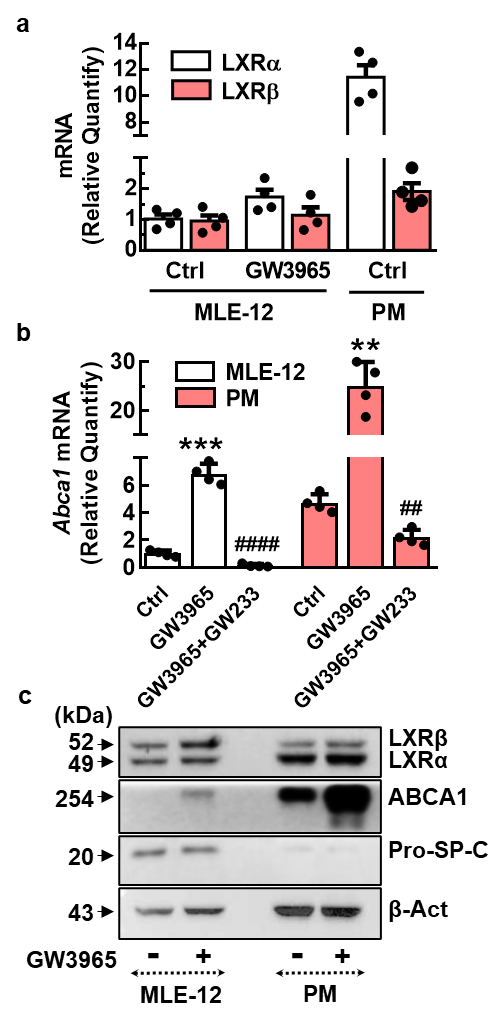
**Fig S4. LXRα and LXRβ are present and active in MLE-12 cell line. a.** MLE-12 cells were incubated in absence (Ctrl) or presence of LXR agonist, GW3965 (GW). Subsequently, transcript levels of LXRα and LXRβ was measured, by real-time qPCR, using peritoneal macrophage (PM) as reference cell. Data represent the mean and standard error of three experiments (n = 4 mice per genotype). **b.** MLE-12 cells and PM were preincubated with LXR agonist GW3965 (GW) or vehicle (Ctrl), in the absence or presence of LXR antagonist GW233. Subsequently, expression of *ABCA1*, was analyzed by real-time qPCR. Data represent the mean and standard error of three experiments (n = 4 mice per genotype). Unpaired Student’s t test with Welch's correction was used for two-group comparisons; ******p < 0.01; *******p < 0.001 compared to Ctrl group; ***^####^****P* < 0.0001 compared to GW group. **c.** MLE-12 cells and PM were incubated in absence (Ctrl) or presence of LXR agonist, GW3965 (GW). Subsequently, expression of LXRα, LXRβ, ABCA1 and pro-SP-C was analyzed by western-blot. Membranes were stripped and reprobed with β−actin antibody as loading control. A representative western blot (n = 3) is shown.

**Table S1**: Antibodies and Dilutions Used in Western Blot (WB), Immunocytochemistry (ICQ), and Immunohistochemistry (IHQ)

| **Antibody** | **Supplier** | **WB** | **ICQ** | **IHQ** |
| --- | --- | --- | --- | --- |
| Rabbit anti-ABCA1 | Sigma | 1:1000 |  |  |
| Mouse anti β-actin | Santa Cruz | 1:200 |  |  |
| Rat anti-B220 | Catlag |  |  | 1:300 |
| Rat anti-CD3 | Hibridoma |  |  | Pure |
| Rat anti-CD68 | Serotec |  | 1:300 | 1:300 |
| Rabbit anti-GFP | Abcam |  |  | 1:5000 |
| Rabbit anti-LXR alpha-beta | Karolinska Institutet (Sweden) | 1:1000 |  |  |
| Rabbit anti- pro-SP-C | Millipore | 1:1000 |  |  |
| Rabbit anti‐Rat biotin | Dako |  |  | 1:150 |
| Goat anti‐rabbit biotin | Dako |  |  | 1:150 |
| Goat anti‐rat IgG-Alexa Fluor ®488 | Invitrogen |  | 1:400 | 1:400 |
| Goat anti‐rat IgG-Alexa Fluor ®594 | Invitrogen |  | 1:500 | 1:500 |
| Goat anti‐mouse HRP | Sta. Cruz | 1:10000 |  |  |
| Goat anti‐rabbit HRP | Sta. Cruz | 1:10000 |  |  |
| Goat anti-mouse IgA | Santa Cruz | 1:1000 |  |  |
| Bovine anti-mouse IgG | Santa Cruz | 1:1000 |  |  |
| Goat anti-mouse IgM | Santa Cruz | 1:1000 |  |  |

**Table S2:** Primers used for qPCR analysis.

| **Mouse Gene** | **Primers** |
| --- | --- |
| ***Abca1* (ABCA1)** | forward (5’ to 3’): GCAGATCAAGCATCCCAACT |
|  | reverse (5’ to 3’): CCAGAGAATGTTTCATTGTCC |
| ***Abca3* (ABCA3)** | forward (5’ to 3’): CCTTCTTCAGTAAAGCCAAC |
|  | reverse (5’ to 3’): CCAGGCCATAGAGTGCTGAG |
| ***Abcg1* (ABCG1)** | forward (5’ to 3’): TCACCC AGTTCTGCATCCTCTT |
|  | reverse (5’ to 3’): GCAGATGTGTCAGGACCGAGT |
| ***Acat1* (ACAT1)** | forward (5’ to 3’): TTTCTCTGGGCCATCCAAT |
|  | reverse (5’ to 3’): AGGGCATGAGCCATATGAAC |
| ***GpaT***  **(GPAT)** | forward (5’ to 3’): GGAAGGTGCTGCTATTCCTG |
|  | reverse (5’ to 3’): TGGGATACTGGGGTTGAAAA |
| ***Lpcat1* (LPCAT1)** | forward (5’ to 3’): GCTGGTGAGCTGTCCTTGAT |
|  | reverse (5’ to 3’): GGTGCACGTTCCTTGAAGA |
| ***Lpcat3* (LPCAT3)** | forward (5’ to 3’): TTCAGTTTGGCATGCTGGT |
|  | reverse (5’ to 3’): ATTGTCCTGGCTCAGGATTC |
| ***Nr1h3***  **(LXRα)** | forward (5’ to 3’): CAACAGTGTAACAGGCGCT |
|  | reverse (5’ to 3’): TGCAATGGGCCAAGGC |
| ***Nr1h2***  **(LXRβ)** | forward (5’ to 3’): CCCCACAAGTTCTCTGGACACT |
|  | reverse (5’ to 3’): TGACGTGGCGGAGGTACTG |
| ***Mip1b***  **(MIP-1β)** | forward (5’ to 3’): CATGAAGCTCTGCGTGTCTG |
|  | reverse (5’ to 3’): GGAGGGTCAGAGCCCATT |
| ***Mmp8***  **(MMP-8)** | forward (5’ to 3’): AACGGGAAGACATACTTCTTCATAA |
|  | reverse (5’ to 3’): GGGTCCATGGATCTTCTTTG |
| ***Mmp12* (MMP-12)** | forward (5’ to 3’): TGCTGATGACATACGTAACATTCA |
|  | reverse (5’ to 3’): TTGTCAAGGATGGGGGTTT |
| ***Sftpa1***  **(SP-A)** | forward (5’ to 3’): CTGGAGAACATGGAGACAAGG |
|  | reverse (5’ to 3’): AAGCTCCTCATCCAGGTAAGC |
| ***Sftpb***  **(SP-B)** | forward (5’ to 3’): AACCCCACACCTCTGAGAAC |
|  | reverse (5’ to 3’): GTGCAGGCTGAGGCTTGT |
| ***Sftpc***  **(SP-C)** | forward (5’ to 3’): GGTCCTGATGGAGAGTCCAC |
|  | reverse (5’ to 3’): GATGAGAAGGCGTTTGAGGT |
| ***Sftpd***  **(SP-D)** | forward (5’ to 3’): GGCCTTAAAAGGAAAACTACAGC |
|  | reverse (5’ to 3’): CCATCAGGGAACAATGCAG |
| ***Spp1***  **(SPP-1)** | forward (5’ to 3’): CCCGGTGAAAGTGACTGATT |
|  | reverse (5’ to 3’): ATCTGGGTGCAGGCTGTAA |
| ***Srebf1* (SREBP-1C)** | forward (5’ to 3’): TGGATCTGTACACTTTACACAGTTCCA |
|  | reverse (5’ to 3’): CCCGGTTTTCTGAAAACTCAAA |
